# Phosphonate as a Stable Zinc‐Binding Group for “Pathoblocker” Inhibitors of Clostridial Collagenase H (ColH)

**DOI:** 10.1002/cmdc.202000994

**Published:** 2021-03-16

**Authors:** Katrin Voos, Esther Schönauer, Alaa Alhayek, Jörg Haupenthal, Anastasia Andreas, Rolf Müller, Rolf W. Hartmann, Hans Brandstetter, Anna K. H. Hirsch, Christian Ducho

**Affiliations:** ^1^ Department of Pharmacy Pharmaceutical and Medicinal Chemistry Saarland University Campus C2 3 66123 Saarbrücken Germany; ^2^ Department of Biosciences and Christian Doppler Laboratory for Innovative Tools for Biosimilar Characterization Division of Structural Biology University of Salzburg Billrothstrasse 11 5020 Salzburg Austria; ^3^ Department of Drug Design and Optimization Helmholtz Institute for Pharmaceutical Research Saarland (HIPS) Helmholtz Centre for Infection Research (HZI) Campus E8 1 66123 Saarbrücken Germany; ^4^ Department of Microbial Natural Products Helmholtz Institute for Pharmaceutical Research Saarland (HIPS) Helmholtz Centre for Infection Research (HZI) Campus E8 1 66123 Saarbrücken Germany; ^5^ Department of Pharmacy Saarland University Campus E8 1 66123 Saarbrücken Germany

**Keywords:** anti-infectives, drug design, medicinal chemistry, metalloenzymes, structure–activity relationships

## Abstract

Microbial infections are a significant threat to public health, and resistance is on the rise, so new antibiotics with novel modes of action are urgently needed. The extracellular zinc metalloprotease collagenase H (ColH) from *Clostridium histolyticum* is a virulence factor that catalyses tissue damage, leading to improved host invasion and colonisation. Besides the major role of ColH in pathogenicity, its extracellular localisation makes it a highly attractive target for the development of new antivirulence agents. Previously, we had found that a highly selective and potent thiol prodrug (with a hydrolytically cleavable thiocarbamate unit) provided efficient ColH inhibition. We now report the synthesis and biological evaluation of a range of zinc‐binding group (ZBG) variants of this thiol‐derived inhibitor, with the mercapto unit being replaced by other zinc ligands. Among these, an analogue with a phosphonate motif as ZBG showed promising activity against ColH, an improved selectivity profile, and significantly higher stability than the thiol reference compound, thus making it an attractive candidate for future drug development.

## Graphical abstract

**Stable “pathoblocker”**: A series of compounds with non‐thiol (stable) zinc‐binding groups has been synthesised and tested for inhibition of the collagenase ColH, a key mediator of clostridial pathogenicity. The most promising compound, a phosphonate, was studied for selectivity over potential human off‐targets and its toxicity both *in vitro* and *in vivo*, and was shown to significantly reduce collagenase activity.

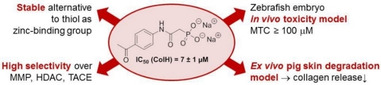

## Introduction

Due to emerging resistances against established antibacterial agents, the treatment of bacterial infections might be thrown back to a state similar to the pre‐antibiotic era. In an estimated worst‐case scenario, it has been predicted that by 2050, infectious diseases caused by antibiotic‐resistant microbes might lead to higher death tolls than cancer does today.^[1]^ Hence, there is an urgent need to develop antibiotics with novel modes of action, high efficacy and a reduced tendency to induce the development of resistances.^[2]^

A promising way of overcoming the problem of fast resistance development is the design of so‐called “pathoblockers”, that is, compounds that target virulence factors rather than vital factors of bacteria, in contrast to classical antibiotics.^[3]^ Bacteria that are thus “disarmed” by pathoblockers should ideally cause either no or at least a strongly attenuated disease. Furthermore, such pathoblocker‐induced reduction of pathogenicity should provide the immune system the necessary time to develop a full humoral and cellular immune response to eliminate the bacteria, possibly aided by a low‐dose adjunctive treatment with antibiotics.

*Clostridium* (including the prominent species *C. difficile*, *C. histolyticum* (*Hathewaya histolytica*), *C. tetani*, *C. botulinum*, *C. septicum*, and *C. perfringens*) is a genus of Gram‐positive anaerobic bacteria that is ubiquitous. They cause severe human diseases such as tetanus, gas gangrene (myonecrosis), botulism, bacterial corneal keratitis, and other dangerous infections^[4]^ with high mortality rates.^[5]^ Some of these species have even been cultivated and are bioweapons.^[6]^

Collagenase is a prominent virulence factor for the progression of Clostridia‐associated diseases.^[7]^ It is a calcium‐ and zinc‐dependent metalloprotease that destroys the host‘s connective tissue and uses it as a carbon source. This leads to improved host invasion and colonisation and hence to breaching of the human immune system. Also, the spread of toxins into the damaged tissue is promoted.[^5b^, ^8^] Collagens, the natural substrates of ColH, are the most abundant proteins of the human extracellular matrix and can be found throughout all organs (especially in skin, bones and joints). Their triple‐helical structure is formed by three intertwined left‐handed helices and is based on a shared repetitive Gly‐X_aa_‐Y_aa_ motif. In this motif, X_aa_ and Y_aa_ can be nearly any amino acid, but a proline in the X_aa_ (28 %) and a hydroxyproline in the Y_aa_ (38 %) position, respectively, occur most frequently.^[10]^

Clostridial collagenases are multidomain proteins whose collagenolytic core is composed of an activator domain and a peptidase domain.^[11]^ In the latter, the catalytic zinc ion is coordinated by two histidines in an HEXXH motif and a downstream glutamate.[^5a^, ^11^] Mechanistically, the general acid‐base glutamate in the HEXXH motif polarises the nucleophilic water molecule in the active site. This polarisation is further facilitated by the zinc ion acting as a Lewis acid (promoted water mechanism). Additionally, by polarising and stabilising the carbonyl oxygen, the zinc ion simultaneously increases the electrophilicity of the carbonyl carbon atom of the scissile amide bond in the bound collagen substrate.^[12]^ Co‐crystal structures of collagenase H (ColH) and collagenase G (ColG) from *C. histolyticum* with a selective and an unselective binder, respectively, are available (PDB IDs: ColH with selective inhibitor: 5O7E;^[13]^ ColG with unselective inhibitor: 2Y6I^[11]^). Clostridial collagenases represent “true” collagenases, that is, they are collagen‐specific and can degrade collagen in its native triple‐helical structure. This cleavage can occur at multiple sites, thus generating small peptide fragments.[^7^, ^8a^] In contrast, human matrix metalloproteases (MMPs), that also include “true” collagenases, are only able to cleave collagen at one site.^[14]^ After cleavage, the collagen fragments then have to undergo further hydrolytic steps catalysed by different enzymes.

**Figure 1:**
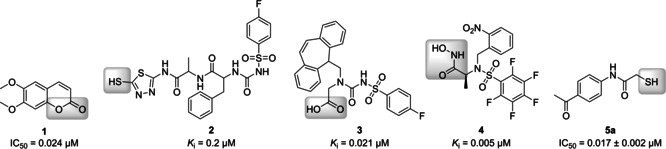
Structural diversity of selected previously reported ColH inhibitors with the zinc‐binding groups highlighted in grey.^[9]^

Besides the crucial role of clostridial collagenases in disease development, their extracellular localisation^[7]^ makes them highly attractive drug targets. The penetration of the bacterial cell wall often represents a major challenge for antibacterial drug development,^[15]^ but can thus be avoided in this case. Naturally occurring collagenase‐inhibiting coumarin derivatives (e. g., **1**, Figure 1) were found in extracts of *Viola yedoensis*.^[9a]^ Supuran and co‐workers have made major contributions to the emerging field of synthetic inhibitors of bacterial collagenases. They have prepared and studied a variety of compounds that mimic the natural substrate with an amide backbone and bind strongly to the active site via a zinc‐binding group (ZBG). The latter coordinates to the zinc ion and displaces the essential water molecule from the active site. The inhibitors vary in their different ZBG, including 2‐mercapto‐substituted 1,3,4‐thiadiazole (e. g., **2**),^[9b]^ carboxylate (e. g., **3**),^[9c]^ and hydroxamic acid units (e. g., **4**;[^9d^, ^9e^, ^9f^] Figure 1). However, all of these compounds show one major drawback in that they are also strong inhibitors of human MMPs due to highly homologous motifs in the active sites of both enzyme families. These off‐target effects severely limit their potential to become suitable drug candidates.

**Scheme 1:**
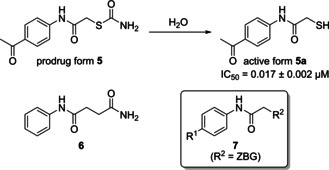
Structures of the previously identified “hit” ColH inhibitor **5**, its active thiol form **5 a** (after prodrug cleavage) and previously reported amide analogue **6**. General structure **7** of the novel potential collagenase inhibitors reported in this work (ZBG=zinc‐binding group).

More recently, we have reported the first selective inhibitor **5** of bacterial collagenases. “Hit” compound **5** contains a thiocarbamate unit as a hydrolytically cleavable prodrug moiety of the thiol **5 a**, which then strongly binds to the zinc ion and therefore inhibits ColH in the low‐nanomolar range (Scheme 1).^[13]^ This compound was also co‐crystallised with the target enzyme, revealing the exact binding mode in the non‐primed binding region. The interactions with the non‐primed edge strand, whose conformation is conserved and distinct for clostridial collagenases, provide the high selectivity towards various bacterial collagenases, including ColH and ColG from *C. histolyticum*, ColT from *C. tetani*, and ColQ1 from *Bacillus cereus*, over the unwanted inhibition of human MMPs.^[13]^

Very recently, we demonstrated that the linker unit in structures of type **5 a**, that is, the motif connecting the aromatic moiety and the thiol ZBG, can be varied. Thus, cyclisation to a succinimide is tolerated with a moderate loss of activity if the thiol is kept in its relative position.^[16]^ This, however, indicated that inhibitor **5 a** can be further varied and optimised. One obvious objective of such an optimisation process would be the replacement of the ZBG. Even though there are thiol‐containing drugs in clinical use,[^17a^, ^17b^] thiols generally suffer from their limited stability, mainly due to oxidative disulfide formation that often leads to rapid inactivation.^[17c]^ Therefore, a replacement of the thiol with a different ZBG would be highly useful, even if it might potentially lead to some loss of inhibitory activity. Our goal was to retain the high selectivity of **5 a** for the inhibition of bacterial collagenases vs. human off‐targets. Hence, the *N*‐arylacetamide core structure was kept intact and only the thiol moiety as the metal‐binding group was exchanged for various other ZBGs. Among the significant number of zinc‐binding units described in the literature,^[18]^ we decided to focus on sterically smaller motifs in order to retain the previously identified binding mode.^[13]^

An amide **6** as a stable analogue of the thiocarbamate structure of prodrug **5** (Scheme 1) has already been reported by us.^[13]^ Amide **6** had the same length as the thiocarbamate **5**. However, it was more than 400‐fold less potent as a ColH inhibitor. This result suggested to us that the overall length of **6** might not fit into the ColH active site, as the (shorter) thiol **5 a** (and not thiocarbamate **5**) was the actual zinc‐binding inhibitor. This consideration has significantly influenced our design of the novel series **7** of potential ColH inhibitors described herein (Scheme 1). Thus, most of the new target compounds contained only one methylene unit to connect the amide carbonyl of the core structure and the ZBG (in analogy to **5 a**). In this work, we therefore report the synthesis and biological evaluation of 16 novel analogues of **5 a** with alternative, more stable ZBGs attached to the core structure (general structure **7**, Scheme 1).

## Results and Discussion

### Chemistry

**Scheme 2:**
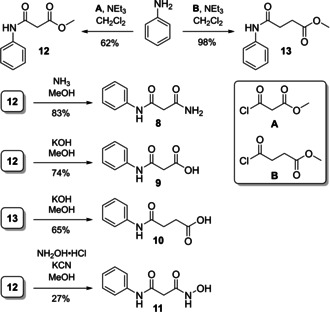
Synthesis of target compounds **8**–**11**.

As a first set of target compounds, we prepared new analogues **8**–**11** with amide (**8**), carboxylate (**9**, **10**) and hydroxamate (**11**) units as potential ZBG (Scheme 2, see also Table 1). Succinic acid derivative **10** is a notable exception to the aforementioned design principle (cf. general structure **7**; Scheme 1) as it was the higher homologue of malonic acid derivative **9** and the acid derivative of amide **6**, thus having one extra methylene unit to connect the ZBG to the core structure.

**Table 1:** *In vitro* inhibitory activities of all synthesised target compounds as well as reference compounds **5 a** and **6** against ColH.

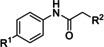											
Cpd.	R^1^	R^2^	IC_50_ [μm]	Cpd.	R^1^	R^2^	IC_50_ [μm]	Cpd.	R^1^	R^2^	IC_50_ [μm]
**5 a**	Ac	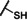	0.017±0.002^[13]^	**14**	Ac	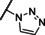	>500	**20**	Ac	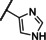	>500
**6**	H	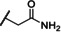	>500^[13]^	**15**	Ac	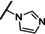	448±33	**23**	Ac	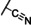	>500
**8**	H	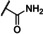	>500	**16**	Ac	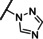	>500	**24**	Ac	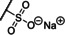	>500
**9**	H	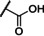	>500	**17**	Ac	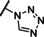	>500	**25**	Ac	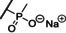	>500
**10**	H	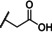	>500	**18**	Ac	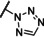	>500	**26**	Ac	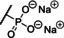	7±1
**11**	H	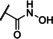	∼500	**19**	Ac	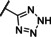	22±1	**27**	Ac	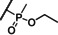	>500

Target compounds **8–11** were obtained via acylation of aniline to give methyl esters **12** and **13** as intermediates in 62 and 98 % yield, respectively. Subsequently, **12** was treated with ammonia, potassium hydroxide or hydroxylamine to furnish **8**, **9** and **11**, respectively, in 27 to 83 % yield. Ester saponification of **13** afforded **10** in 65 % yield (Scheme 2).

**Scheme 3:**
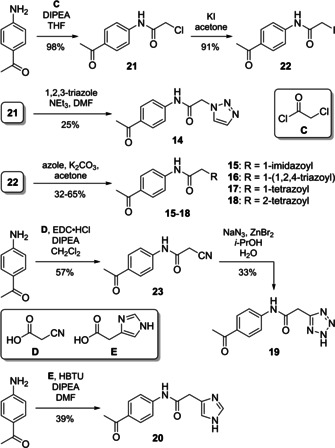
Synthesis of azole‐derived target compounds **14**–**20**.

The zinc ion in the active site of ColH is complexed by two histidine residues.[^5a^, ^11^, ^13^] Therefore, azole compounds **14**–**20** (Scheme 3, see also Table 1) were synthesised to mimic the imidazole moiety of histidine. Triazole derivative **14** was prepared in the following manner. Acylation of *p*‐aminoacetophenone with chloroacetyl chloride gave alkyl chloride **21** in 98 % yield, and alkylation of 1,2,3‐triazole with **21** furnished **14** in 25 % yield. In order to obtain an alkylating agent with higher reactivity, **21** was converted into alkyl iodide **22** in a Finkelstein reaction (91 % yield). Iodide **22** was then used for the alkylation of other azoles, affording target compounds **15**–**18** in 32–65 % yield. Amide coupling of *p*‐aminoacetophenone and cyanoacetic acid gave nitrile intermediate **23** in 57 % yield, which was transformed into the target tetrazole **19** by zinc‐catalysed cycloaddition with sodium azide (33 % yield). Imidazole‐derived analogue **20** was synthesised in one step (by amide coupling of *p*‐aminoacetophenone) in 39 % yield (Scheme 3).

**Scheme 4:**
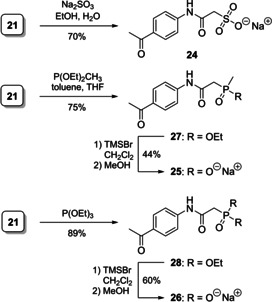
Synthesis of anionic target compounds **24**–**26**.

In order to exploit the potential of electrostatic interactions, negatively charged ZBGs were also explored. Hence, target compounds **24** (with a sulfonate unit as ZBG), **25** (phosphinate), and **26** (phosphonate) were synthesised using the previously employed alkyl chloride intermediate **21** (Scheme 4). Sulfonate **24** was obtained by alkylation of sulfite in 70 % yield. Both phosphinate **25** and phosphonate **26** were prepared in two steps each, with the first step being a Michaelis–Arbuzov reaction to give ethyl esters **27** and **28** in 75 and 89 % yield, respectively. This was followed by silyl‐mediated cleavage of the ethyl ester to afford (after ion exchange) highly pure sodium salts **25** and **26** in yields of 44 and 60 %, respectively.

### *In vitro* inhibition of ColH

All synthesised target compounds were tested in a previously described FRET‐based *in vitro* assay for ColH inhibition, using a custom‐made quenched fluorescent peptide as substrate.^[13]^ The inhibitory activities (IC_50_ values) were obtained from steady‐state kinetics. Varying concentrations of the compounds were preincubated with the peptidase domain of ColH (10 nm) for 1 h before the reactions were started by the addition of the peptide substrate. IC_50_ values were determined by nonlinear regression analysis and are listed in Table 1.

Interestingly, only two out of the 16 tested compounds with various zinc‐binding groups showed notable inhibitory activity against ColH. These were tetrazole derivative **19** (IC_50_=22 μm) and phosphonate **26** (IC_50_=7 μm). However, both compounds were considerably weaker inhibitors of ColH than thiol **5 a** (IC_50_=17 nm). As **26** was about three times more active than **19**, it was decided to further investigate phosphonate **26** for other biological properties in order to elucidate if it might be a suitable alternative to **5 a**, in spite of its decreased inhibitory potency towards the target collagenase ColH.

### Selectivity over potential human off‐targets

Inhibitors of bacterial collagenases might potentially also bind to human zinc‐dependent enzymes as “off‐targets”, even though the results obtained with thiocarbamate **5** had demonstrated that pronounced selectivity can be achieved (see above).^[13]^ We have therefore investigated if phosphonate **26** (in comparison to thiol **5 a** as the active form of **5**) inhibited a selection of such zinc‐dependent human off‐targets. Our goal was to at least retain the selectivity of “hit” compound **5** when the ZBG is changed. The human enzymes chosen for this study were six representatives of the MMP family, histone deacetylases 3 (HDAC‐3) and 8 (HDAC‐8), and the tumour necrosis factor α (TNF‐α) converting enzyme (TACE, also known as ADAM‐17). It should be noted that **5** had previously only been tested for the unwanted inhibition of MMPs.^[13]^

Inhibitory activities of both **5 a** and novel ColH inhibitor **26** against this panel of potential human off‐targets are provided in Table 2 (as percentage inhibition at a fixed concentration of 100 μm). Against the six selected MMP enzymes, both ColH inhibitors showed no notable to very moderate inhibition at this rather high concentration. An exception was the inhibition of MMP‐8 (36 % @100 μm) and MMP‐14 (47 % @100 μm), respectively, by phosphonate **26**. In contrast, both enzymes were not inhibited by thiol **5 a**.^[13]^ However, this implies that the *in vitro* inhibitory activities of **26** towards these two representatives of the MMP family were still more than one order of magnitude lower than towards ColH as its bacterial target. Overall, the rather limited inhibition of MMPs as human off‐targets by **26** confirmed our initial design principle to retain the aromatic anilide core structure that had been shown to be crucial for the selectivity of **5**/**5 a**.^[13]^

**Table 2:** *In vitro* inhibitory activities of novel ColH inhibitor **26** (ZBG=phosphonate) and reference compound **5 a** (ZBG=thiol) against a panel of potential human off‐targets.

Human enzyme	Inhibition [%] at 100 μm compound^[a]^	
	**5 a** (thiol)	**26** (phosphonate)
MMP‐1	n.i.^[b]^	19±4
MMP‐2	19±8	18±1
MMP‐3	n.i.	n.i.
MMP‐7	n.i.	n.i.
MMP‐8	n.i.	36±1
MMP‐14	n.i.	47±13
HDAC‐3	51±7	11±2
HDAC‐8	48±5	n.i.
TACE	79±7	21±8

[a] Means of at least two independent measurements, 10 nm enzyme concentration. [b] n.i.=no inhibition (<10 %).

As noted, the other potential human off‐targets investigated in this context were two representatives of the HDAC family and TACE. Regarding the unwanted inhibition of these three enzymes, phosphonate **26** was superior to thiol **5 a** throughout (Table 2). Thus, thiol **5 a** showed significantly higher inhibition of the two HDAC tested (∼50 % inhibition with **5 a** vs. maximum ∼10 % with **26**) and in particular of TACE (∼80 % with **5 a** vs. ∼20 % with **26**). The unwanted inhibition of TACE would lead to a decreased release of TNF‐α, hence causing a reduced immune response of the host^[19]^ and thereby providing the bacteria with a higher chance to establish a critical infection.^[20]^ The absence of notable HDAC and TACE inhibition for **26** represents a major advantage of this novel phosphonate‐derived ColH inhibitor.

### Cytotoxicity against human cells

The new ColH inhibitor **26** was also investigated for potential cytotoxic effects against three representative human cell lines, that is, HepG2, HEK293, and A549 cells. Within the experimental error, **26** showed a comparable or even slightly lower decrease of cell viability (as an indicator of toxicity) than the previous “hit” compound **5 a** in all investigated cell lines at 100 μm compound concentration (Table 3). As a reference compound, the approved antibiotic rifampicin (that is clinically used in the long‐term therapy of tuberculosis^[21]^) was also studied. Rifampicin showed a comparable decrease of cell viability as both tested ColH inhibitors **26** and **5 a** at an identical concentration (100 μm). As further references and also as positive controls, the chemotherapeutic agents doxorubicin^[22a]^ and epirubicin^[22b]^ were employed and were found to be notably toxic at a 100‐fold lower concentration (i. e., 1 μm) in all three cell lines. In turn, it can be concluded that even a 100‐fold higher concentration of **26** might be tolerated based on these cytotoxicity studies.

**Table 3:** Cytotoxicity (as decrease of cell viability) of ColH inhibitors **26** (ZBG=phosphonate) and **5 a** (ZBG=thiol) as well as of three reference compounds against three human cell lines.

Compound	*c* [μm]	Decrease of viability [%] after 48 h^[a]^		
		HepG2	HEK293	A549
**5 a** (thiol)	100	15±9	53±0	18±12
**26** (phosphonate)	100	8±10	32±4	20±2
rifampicin	100	33±13	29±13	9±13
doxorubicin	1	57±14	47±9	53±13
epirubicin	1	68±10	49±11	56±12

[a] Means of at least two independent measurements.

### Toxicity in a zebrafish model

In order to determine the toxicity of ColH inhibitors **26** and **5 a** in an *in vivo* setting, a zebrafish larvae toxicity assay was performed. Zebrafish are very small and almost transparent organisms. They can be cultured in small volumes of media, leading to very small amounts of compounds being needed for testing. Furthermore, zebrafish develop their organ systems, which show high similarity to the mammalian cardiovascular, nervous, and digestive systems,[^23a^, ^23b^] in less than one week,[^23a^, ^23c^, ^23d^] thus making the experiments fast and relatively inexpensive. Using this assay, we aimed to determine the maximally tolerated dose (MTD) at which no toxic effects of the compounds were observed. It was found that both phosphonate **26** and thiol reference **5 a** showed no toxic effects at concentrations up to 100 μm.

### *Ex vivo* pig skin degradation assay

**Figure 2:**
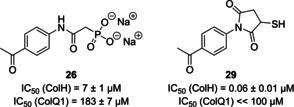
Structures and *in vitro* potencies for collagenase inhibition of compounds **26** and **29**.^[16]^

**Figure 3:**
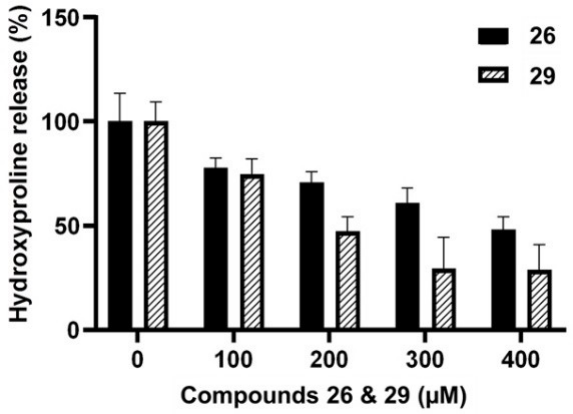
*Ex vivo* pig skin degradation assay: hydroxyproline release after 24 h upon treatment with **26** and **29** ^[16]^ (in % relative to the controls).

To investigate the activity of collagenase inhibitors on tissue in a more complex experimental setting, an *ex vivo* pig skin degradation assay using purified ColQ1 from *B. cereus* has recently been developed.^[16]^ The degradation of collagen in this mammalian tissue was measured as a rate of hydroxyproline (Hyp) release. This assay had previously been used to demonstrate the efficacy of succinimide **29** (Figure 2) as an inhibitor of bacterial collagenase activity.^[16]^ Succinimide derivative **29** had an IC_50_ value of 0.06±0.01 μm against ColH *in vitro*, which indicates that it is ca. 100‐fold more potent than phosphonate **26**. Towards ColQ1 (at a fixed inhibitor concentration of 100 μm), **29** had shown complete (100±2%) inhibition, which corresponded to an IC_50_ value significantly below 100 μm.^[16]^ Using the same *in vitro* assay, novel phosphonate **26** had an IC_50_ value of 183±7 μm. However, in the *ex vivo* assay, **26** reduced the formation of Hyp to 75 % of the Hyp production of the control at a concentration of 100 μm, which is nearly identical to the potency of **29** (Figure 3).

Overall, succinimide **29** showed a stronger reduction of Hyp formation at elevated concentrations than phosphonate **26**, in particular at 300 and 400 μm, respectively (Figure 3). However, this difference in potency was far less than what was expected based on the significant difference of the aforementioned *in vitro* activities (IC_50_ values). It should also be noted that succinimide **29** appears to reach a “plateau” in activity between about 300 and 400 μm while phosphonate **26** had a nearly linear correlation of concentration and activity. This “plateau” in the activity of **29** might eventually result from disulfide formation at higher concentrations. This again highlights the benefit of employing an oxidation‐resistant ZBG (as in **26**) to provide air‐stable alternatives to thiol‐derived inhibitors.

## Conclusions

In summary, we herein report the exchange of the thiol ZBG of previously published selective ColH inhibitors with more stable ZBGs, with the selectivity of the thiol‐based “hit” compound **5 a** being retained. Thus, the structure of **5 a** was varied to furnish phosphonate derivative **26**. In contrast to the thiol group in **5 a**, the phosphonate as a ZBG in **26** is not prone to oxidation or degradation. Novel inhibitor **26** still showed reasonably potent (i. e., micromolar) inhibition of the clostridial collagenase ColH, while apparently retaining the binding mode of **5 a** and therefore its remarkable selectivity over potential human off‐targets such as MMPs. In comparison to **5 a**, we observed a better selectivity of **26** over other human off‐targets (HDAC and TACE), suggesting it might be an improved hit structure for further development. Inhibitor **26** showed no indication of major toxicity in three different human cell lines as well as high tolerance in an *in vivo* zebrafish toxicity assay. Furthermore, we demonstrated the efficacy of **26** not only in an *in vitro* enzyme assay for collagenase activity, but also in a more complex *ex vivo* pig skin degradation assay. The results from this assay highlight the potency of **26** and the relevance of a stable (non‐thiol) ZBG for biological activity in a complex biological setting. Overall, **26** should therefore be considered as a new improved hit structure for the development of potent inhibitors of bacterial collagenases that will now undergo further optimisation in our laboratories.

## Experimental Section

**General methods**: All chemicals, starting materials and reagents were purchased from standard suppliers and used without further purification. Air‐ and/or water‐sensitive reactions were carried out under nitrogen atmosphere with anhydrous solvents. Anhydrous solvents were obtained in the following manner: THF, DMF and CH_2_Cl_2_ were dried with a solvent purification system (MBRAUN MB SPS 800). All other solvents were of technical quality and distilled prior to use, and deionised water was used throughout. Reactions were monitored by TLC on aluminium plates precoated with silica gel 60 F254 (VWR). Visualisation of the spots was carried out using UV light (254 nm) and/or staining under heating (VSS stain: 4 g vanillin, 25 mL conc. H_2_SO_4_, 80 mL AcOH, 680 mL MeOH; CAM stain: 12 g ammonium molybdate, 0.5 g ceric ammonium molybdate, 235 mL H_2_O, 15 mL conc. H_2_SO_4_; ninhydrin stain: 1.5 g ninhydrin, 100 mL *n*‐butanol, 3.0 mL AcOH). *R*_f_ values are given to the nearest 0.05. Column chromatography was carried out on silica gel 60 (40–63 μm, 230–400 mesh ASTM, VWR) under flash conditions. Preparative centrifugal TLC was performed on a Chromatotron^TM^ 7924T (T‐Squared Technology) using glass plates coated with silica gel 60 PF_254_ containing a fluorescent indicator (VWR, thickness depending on the amount of crude material to be separated, for 50–500 mg: 1 mm layer). Ion‐exchange chromatography was carried out using DOWEX™ 50WX8 resin (200–400 mesh, VWR) in the Na^+^ form. Semipreparative HPLC was performed on a VWR‐Hitachi system equipped with an L‐2300 pump, an L‐2200 autosampler, an L‐2455 diode array detector (DAD) and a LichroCart^TM^ Purospher^TM^ RP18e column (5 μm, 10×250 mm, VWR). NMR spectra were recorded using the following Bruker NMR spectrometers: for ^1^H NMR spectra at 500 MHz and ^13^C NMR spectra at 126 MHz: Avance™ 500; for ^31^P NMR spectra at 203 MHz: Avance™ 500. For the assignment of signals, ^1^H,^1^H COSY, ^1^H,^13^C HSQC and ^1^H,^13^C HMBC spectra were used. All ^13^C and ^31^P NMR spectra are ^1^H‐decoupled. All spectra were recorded at room temperature if not indicated otherwise and were referenced internally to solvent residual signals wherever possible. Chemical shifts (*δ*) are quoted in ppm and coupling constants (*J*) are reported in Hz. Low‐resolution mass spectra were recorded on a liquid chromatography‐coupled mass spectrometer (LC–MS) Surveyor MSQ Plus from Finnigan. For the LC separation prior to detection, a Nucleodur^TM^ 100–5 C_18_ column (5 μm, 3×125 mm) was used. High‐resolution mass spectra were recorded on a Thermo Scientific Q Exactive Orbitrap mass spectrometer with ESI ionisation mode coupled with an Ultimate 3000 HPLC system by Thermo Scientific, equipped with a Thermo Accucore^TM^ phenyl‐X column (2.1 μm, 3×100 mm). Melting points (mp) were measured on a melting point apparatus SMP3 (Stuart Scientific) and were not corrected.

**General procedure (GP1) for the synthesis of azole derivatives**: Alkyl iodide **22** (1.0 equiv), K_2_CO_3_ (1.1 equiv), and the respective azole (1.1 equiv) were suspended in acetone (20 mL, ∼25 mm) and stirred at 70 °C overnight (15–20 h). EtOAc (200 mL) was added, and the organic layer was washed with water (3×30 mL) and brine (50 mL) and dried over Na_2_SO_4_. The solvent was evaporated under reduced pressure and the resultant crude product was purified by column chromatography to give the respective azole derivative.

**General procedure (GP2) for the cleavage of ethyl esters**: The respective ethyl ester (1.0 equiv) was dissolved in CH_2_Cl_2_ (10 mL). TMSBr (5.0 equiv) was added dropwise over 10 min, and the reaction was stirred at RT overnight (15–20 h). MeOH (10 mL) was then added, and the mixture was stirred for 1–2 h. The solvent was evaporated under reduced pressure, the resultant crude product was purified by HPLC and the obtained product was converted into its Na^+^ form by ion‐exchange column chromatography to give the respective title compound.

***N*** ^**1**^ **‐Phenylmalonamide (8)**: Methyl ester **12** (49 mg, 0.25 mmol) was dissolved in MeOH (6 mL). NH_3_ solution (33 %, 3 mL) was added, and the reaction mixture was stirred at RT overnight. The solvent was evaporated under reduced pressure and the resultant crude product was purified by column chromatography (CH_2_Cl_2_/MeOH 100:0→95 : 5) to give **8** as a white solid (37 mg, 83 %). ^1^H NMR (500 MHz, [D_6_]DMSO): *δ*=10.07 (s, 1H, Ph‐NH), 7.58 (d, *J*=7.6 Hz, 2H, 2′‐H, 6′‐H), 7.51 (br, 1H, NH_2_‐a), 7.30 (t, *J*=7.9 Hz, 2H, 3′‐H, 5′‐H), 7.11 (br, 1H, NH_2_‐b), 7.04 (t, *J*=7.4 Hz, 1H, 4′‐H), 3.21 (s, 2H, 2‐H) ppm. ^13^C NMR (126 MHz, [D_6_]DMSO): *δ*=168.68 (C‐1 or C‐3), 165.74 (C‐1 or C‐3), 138.96 (C‐1′), 128.71 (C‐3′, C‐5′), 123.27 (C‐4′), 119.02 (C‐2′, C‐6′), 44.50 (C‐2) ppm. HRMS (ESI): calcd. for C_9_H_11_N_2_O_2_ [*M*+H]^+^ 179.0815, found 179.0813. TLC (petroleum ether/EtOAc 1 : 1): *R*_f_=0.10.

**3‐Oxo‐3‐(phenylamino)propanoic acid (9)**: Methyl ester **12** (102 mg, 0.528 mmol) was dissolved in MeOH (5 mL). KOH solution (50 g/L, 1.2 mL) was added, and the reaction mixture was stirred at 35 °C for 4 h. It was then acidified with HCl, and EtOAc (200 mL) was added. The organic layer was washed with HCl (2 m, 4×40 mL) and brine (50 mL) and then dried over Na_2_SO_4_. The solvent was evaporated under reduced pressure, and the resultant crude product was purified by column chromatography (CH_2_Cl_2_/MeOH/HCOOH 95 : 4 : 1) to give **9** as a white solid (70 mg, 74 %). ^1^H NMR (500 MHz, [D_6_]DMSO): *δ*=12.68 (br, 1H, COOH), 10.13 (s, 1H, NH), 7.57 (d, *J*=7.6 Hz, 2H, 2′‐H, 6′‐H), 7.31 (t, *J*=7.9 Hz, 2H, 3′‐H, 5′‐H), 7.05 (t, *J*=7.4 Hz, 1H, 4′‐H), 3.34 (s, 2H, 2‐H) ppm. ^13^C NMR (126 MHz, [D_6_]DMSO): *δ*=169.28 (C‐1 or C‐3), 164.57 (C‐1 or C‐3), 138.95 (C‐1′), 128.76 (C‐3′, C‐5′), 123.38 (C‐4′), 119.01 (C‐2′, C‐6′), 43.99 (C‐2) ppm. HRMS (ESI): calcd. for C_9_H_10_NO_3_ [*M*+H]^+^ 180.0655, found 180.0654. TLC (petroleum ether/EtOAc/HCOOH 49 : 49 : 2): *R*_f_=0.20.

**4‐Oxo‐4‐(phenylamino)butanoic acid (10)**: Methyl ester **13** (204 mg, 0.985 mmol) was dissolved in MeOH (4 mL). KOH solution (50 g/L, 2.2 mL) was added, and the reaction mixture was stirred at 35 °C for 4 h. It was then acidified with HCl and EtOAc (200 mL) was added. The organic layer was washed with HCl (2 m, 4×40 mL) and brine (50 mL) and then dried over Na_2_SO_4_. The solvent was evaporated under reduced pressure, and the resultant crude product was purified by column chromatography (petroleum ether/EtOAc/HCOOH 70 : 29 : 1) to give **10** as a white solid (124 mg, 65 %). ^1^H NMR (500 MHz, [D_6_]DMSO): *δ*=12.20 (br, 1H, COOH), 9.94 (br, 1H, NH), 7.57 (d, *J*=7.6 Hz, 2H, 2′‐H, 6′‐H), 7.28 (t, *J*=7.9 Hz, 2H, 3′‐H, 5′‐H), 7.01 (t, *J*=7.4 Hz, 1H, 4′‐H), 2.56‐2.50 (m, 4H, 2‐H, 3‐H) ppm. ^13^C NMR (126 MHz, [D_6_]DMSO): *δ*=173.83 (C‐1 or C‐4), 170.06 (C‐1 or C‐4), 139.30 (C‐1′), 128.65 (C‐3′, C‐5′), 122.88 (C‐4′), 118.87 (C‐2′, C‐6′), 31.04 (C‐2 or C‐3), 28.82 (C‐2 or C‐3) ppm. HRMS (ESI): calcd. for C_10_H_12_NO_3_ [*M*+H]^+^ 194.0812, found 194.0810. TLC (petroleum ether/EtOAc/HCOOH 49 : 49 : 2): *R*_f_=0.25.

***N*** ^**1**^ **‐Hydroxy‐*N*** ^**3**^ **‐phenylmalonamide (11)**: Hydroxylamine hydrochloride (497 mg, 7.15 mmol), DIPEA (1.50 mL, 8.61 mmol) and KCN (19 mg, 0.29 mmol) were dissolved in MeOH (5 mL), and the mixture was heated to reflux. After 10 min, a solution of methyl ester **12** (91 mg, 0.47 mmol) in MeOH (5 mL) was added, and the reaction mixture was stirred under reflux overnight. It was then concentrated under reduced pressure, acidified with HCl (1 m, 100 mL) and extracted with EtOAc (5×50 mL). The combined organics were dried over Na_2_SO_4_, and the solvent was evaporated under reduced pressure. The resultant crude product was purified by HPLC (water+0.1 % TFA, MeCN+0.1 % TFA, 95 : 5→0 : 100) to give **11** as a slightly orange solid (25 mg, 27 %). ^1^H NMR (500 MHz, [D_6_]DMSO): *δ*=10.61 (s, 1H, NHOH), 10.10 (s, 1H, Ph‐NH), 8.96 (br, 1H, NHOH), 7.57 (d, *J*=7.8 Hz, 2H, 2′‐H, 6′‐H), 7.30 (t, *J*=7.8 Hz, 2H, 3′‐H, 5′‐H), 7.05 (t, *J*=7.3 Hz, 1H, 4′‐H), 3.11 (s, 2H, 2‐H) ppm. ^13^C NMR (126 MHz, [D_6_]DMSO): *δ*=165.27 (C‐1 or C‐3), 163.48 (C‐1 or C‐3), 138.93 (C‐1′), 128.76 (C‐3′, C‐5′), 123.38 (C‐4′), 119.07 (C‐2′, C‐6′), 42.03 (C‐2) ppm. HRMS (ESI): calcd. for C_9_H_9_N_2_O_3_ [*M*−H]^−^ 193.0619, found: 193.0609. TLC (CH_2_Cl_2_/MeOH/HCOOH 90 : 9 : 1): *R*_f_=0.15.

**Methyl 3‐oxo‐3‐(phenylamino)propanoate (12)**: Aniline (400 μL, 4.39 mmol) and NEt_3_ (1.20 mL, 8.61 mmol) were dissolved in CH_2_Cl_2_ (10 mL) and cooled to 0 °C. Methyl malonyl chloride (500 μL, 5.24 mmol) was added dropwise over 15 min, and the reaction mixture was stirred at 0 °C for 3 h. The reaction was quenched with cold water (15 mL), and the mixture was diluted with CH_2_Cl_2_. The organic layer was washed with sat. NaHCO_3_ solution (5×40 mL) and dried over Na_2_SO_4_. The solvent was evaporated under reduced pressure, and the resultant crude product was purified by column chromatography (CH_2_Cl_2_) to give **12** as a slightly orange solid (523 mg, 62 %). ^1^H NMR (500 MHz, CDCl_3_): *δ*=9.15 (br, 1H, NH), 7.55 (d, *J*=8.1 Hz, 2H, 2′‐H, 6′‐H), 7.33 (t, *J*=7.9 Hz, 2H, 3′‐H, 5′‐H), 7.13 (t, *J*=7.4 Hz, 1H, 4′‐H), 3.80 (s, 3H, OCH_3_), 3.49 (s, 2H, 2‐H) ppm. ^13^C NMR (126 MHz, CDCl_3_): *δ*=170.58 (C‐1 or C‐3), 162.82 (C‐1 or C‐3), 137.54 (C‐1′), 129.15 (C‐3′, C‐5′), 124.76 (C‐4′), 120.25 (C‐2′, C‐6′), 52.82 (OCH_3_), 41.45 (C‐2) ppm. MS (ESI): *m/z*=216.0 [*M*+Na]^+^. TLC (petroleum ether/EtOAc 7 : 3): *R*_f_=0.10.

**Methyl 4‐oxo‐4‐(phenylamino)butanoate (13)**: Aniline (100 μL, 1.10 mmol) and NEt_3_ (300 μL, 2.15 mmol) were dissolved in CH_2_Cl_2_ (5 mL) and cooled to 0 °C. Methyl succinyl chloride (170 μL, 1.38 mmol) was added dropwise over 10 min, and the reaction mixture was stirred at 0 °C for 3.5 h. The reaction was quenched with cold water (15 mL), and the mixture was diluted with CH_2_Cl_2_. The organic layer was washed with sat. NaHCO_3_ solution (4×30 mL) and dried over Na_2_SO_4_. The solvent was evaporated under reduced pressure and the resultant crude product was purified by centrifugal TLC (CH_2_Cl_2_) to give **13** as a white solid (222 mg, 98 %). ^1^H NMR (500 MHz, CDCl_3_): *δ*=7.71 (br, 1H, NH), 7.50 (d, *J*=7.9 Hz, 2H, 2′‐H, 6′‐H), 7.29 (t, *J*=7.9 Hz, 2H, 3′‐H, 5′‐H), 7.09 (t, *J*=7.4 Hz, 1H, 4′‐H), 3.70 (s, 3H, OCH_3_), 2.75 (t, *J*=6.5 Hz, 2H, 2‐H or 3‐H), 2.66 (t, *J*=6.5 Hz, 2H, 2‐H or 3‐H) ppm. ^13^C NMR (126 MHz, CDCl_3_): *δ*=173.78 (C‐1 or C‐4), 169.84 (C‐1 or C‐4), 137.97 (C‐1′), 129.07 (C‐3′, C‐5′), 124.35 (C‐4′), 119.90 (C‐2′, C‐6′), 52.12 (OCH_3_), 32.20 (C‐2 or C‐3), 29.36 (C‐2 or C‐3) ppm. HRMS (ESI): calcd. for C_11_H_14_NO_3_ [*M*+H]^+^ 208.0968, found 208.0961. TLC (petroleum ether/EtOAc 7 : 3): *R*_f_=0.10.

***N*** **‐(4‐Acetylphenyl)‐2‐(1*H*‐1,2,3‐triazol‐1‐yl)acetamide (14)**: Alkyl chloride **21** (60 mg, 0.28 mmol), NEt_3_ (90 μL, 0.65 mmol) and 1*H*‐1,2,3‐triazole (20 μL, 0.34 mmol) were dissolved in DMF (10 mL) and stirred at RT for 8 d. EtOAc (250 mL) was added, and the organic layer was washed with water (5×30 mL) and brine (30 mL) and then dried over Na_2_SO_4_. The solvent was evaporated under reduced pressure, and the resultant crude product was purified by column chromatography (CH_2_Cl_2_/MeOH 99 : 1) to give **14** as a white solid (17 mg, 25 %). ^1^H NMR (500 MHz, [D_6_]DMSO): *δ*=10.83 (s, 1H, NH), 8.17 (d, *J*=0.9 Hz, 1H, 5′′‐H), 7.96 (d, *J*=8.8 Hz, 2H, 3′‐H, 5′‐H), 7.77 (d, *J*=0.8 Hz, 1H, 4′′‐H), 7.72 (d, *J*=8.8 Hz, 2H, 2′‐H, 6′‐H), 5.41 (s, 2H, 2‐H), 2.53 (s, 3H, acetyl‐CH_3_) ppm. ^13^C NMR (126 MHz, [D_6_]DMSO): *δ*=196.52 (acetyl‐C=O), 164.96 (C‐1), 142.69 (C‐1′), 133.15 (C‐4′′), 132.15 (C‐4′), 129.61 (C‐3′, C‐5′), 126.55 (C‐5′′), 118.53 (C‐2′, C‐6′), 52.04 (C‐2), 26.45 (acetyl‐CH_3_) ppm. MS (ESI): *m/z*=245.0 [*M*+H]^+^. HRMS (ESI): calcd. for C_12_H_13_N_4_O_2_ [*M*+H]^+^ 245.1033, found 245.1030. TLC (CH_2_Cl_2_/MeOH 95 : 5): *R*_f_=0.15.

***N*** **‐(4‐Acetylphenyl)‐2‐(1*H*‐imidazol‐1‐yl)acetamide (15)**: General procedure **GP1** with 1*H*‐imidazole (41 mg, 0.60 mmol) and alkyl iodide **22** (156 mg, 0.515 mmol) to give **15** as a white solid (47 mg, 38 %). ^1^H NMR (500 MHz, [D_6_]DMSO): *δ*=10.65 (s, 1H, NH), 7.95 (d, *J*=8.8 Hz, 2H, 3′‐H, 5′‐H), 7.72 (d, *J*=8.8 Hz, 2H, 2′‐H, 6′‐H), 7.64 (s, 1H, 2′′‐H), 7.17 (t, *J*=1.9 Hz, 1H, 5′′‐H), 6.90 (t, *J*=0.9 Hz, 1H, 4′′‐H), 4.96 (s, 2H, 2‐H), 2.53 (s, 3H, acetyl‐CH_3_) ppm. ^13^C NMR (126 MHz, [D_6_]DMSO): *δ*=196.50 (acetyl‐C=O), 166.43 (C‐1), 142.93 (C‐1′), 138.35 (C‐2′′), 131.93 (C‐4′), 129.59 (C‐3′, C‐5′), 127.92 (C‐4′′), 120.76 (C‐5′′), 118.39 (C‐2′, C‐6′), 49.18 (C‐2), 26.43 (acetyl‐CH_3_) ppm. HRMS (ESI): calcd. for C_13_H_14_N_3_O_2_ [*M*+H]^+^ 244.1081, found 244.1078. TLC (CH_2_Cl_2_/MeOH 9 : 1): *R*_f_=0.20.

***N*** **‐(4‐Acetylphenyl)‐2‐(1*H*‐1,2,4‐triazol‐1‐yl)acetamide (16)**: General procedure **GP1** with 1*H*‐1,2,4‐triazol (46 mg, 0.67 mmol) and alkyl iodide **22** (165 mg, 0.545 mmol) to give **16** as a slightly orange solid (87 mg, 65 %). ^1^H NMR (500 MHz, [D_6_]DMSO): *δ*=10.76 (s, 1H, NH), 8.56 (s, 1H, 5′′‐H), 8.01 (s, 1H, 3′′‐H), 7.95 (d, *J*=8.8 Hz, 2H, 3′‐H, 5′‐H), 7.71 (d, *J*=8.8 Hz, 2H, 2′‐H, 6′‐H), 5.19 (s, 2H, 2‐H), 2.53 (s, 3H, acetyl‐CH_3_) ppm. ^13^C NMR (126 MHz, [D_6_]DMSO): *δ*=196.58 (acetyl‐C=O), 165.26 (C‐1), 151.47 (C‐3′′), 145.69 (C‐5′′), 142.73 (C‐1′), 132.16 (C‐4′), 129.66 (C‐3′, C‐5′), 118.55 (C‐2′, C‐6′), 51.85 (C‐2), 26.51 (acetyl‐CH_3_) ppm. MS (ESI): *m/z*=245.0 [*M*+H]^+^. HRMS (ESI): calcd. for C_12_H_13_N_4_O_2_ [*M*+H]^+^ 245.1033, found 245.1030. TLC (CH_2_Cl_2_/MeOH 95 : 5): *R*_f_=0.10.

***N*** **‐(4‐Acetylphenyl)‐2‐(1*H*‐tetrazol‐1‐yl)acetamide (17) and** ***N*** **‐(4‐acetylphenyl)‐2‐(2*H*‐tetrazol‐2‐yl)acetamide (18)**: General procedure **GP1** with 1*H*‐tetrazole (27 mg, 0.39 mmol) and alkyl iodide **22** (101 mg, 0.333 mmol) to give **17** as a white solid (28 mg, 34 %) and **18** as a white solid (26 mg, 32 %). **17**: ^1^H NMR (500 MHz, [D_6_]DMSO): *δ*=10.90 (s, 1H, NH), 9.43 (s, 1H, 5′′‐H), 7.96 (d, *J*=8.8 Hz, 2H, 3′‐H, 5′‐H), 7.71 (d, *J*=8.8 Hz, 2H, 2′‐H, 6′‐H), 5.54 (s, 2H, 2‐H), 2.53 (s, 3H, acetyl‐CH_3_) ppm. ^13^C NMR (126 MHz, [D_6_]DMSO): *δ*=196.55 (acetyl‐C=O), 164.14 (C‐1), 145.26 (C‐5′′), 142.50 (C‐1′), 132.27 (C‐4′), 129.64 (C‐3′, C‐5′), 118.59 (C‐2′, C‐6′), 50.14 (C‐2), 26.46 (acetyl‐CH_3_) ppm. MS (ESI): *m/z*=246.0 [*M*+H]^+^, 268.0 [*M*+Na]^+^. HRMS (ESI): calcd. for C_11_H_13_N_5_O_2_ [*M*+H]^+^ 246.0986, found 246.0982. TLC (CH_2_Cl_2_/MeOH 95 : 5): *R*_f_=0.25. **18**: ^1^H NMR (500 MHz, [D_6_]DMSO): *δ*=10.95 (s, 1H, NH), 9.05 (s, 1H, 5′′‐H), 7.96 (d, *J*=8.8 Hz, 2H, 3′‐H, 5′‐H), 7.70 (d, *J*=8.8 Hz, 2H, 2′‐H, 6′‐H), 5.78 (s, 2H, 2‐H), 2.53 (s, 3H, acetyl‐CH_3_) ppm. ^13^C NMR (126 MHz, [D_6_]DMSO): *δ*=196.53 (acetyl‐C=O), 163.52 (C‐1), 153.42 (C‐5′′), 142.40 (C‐1′), 132.34 (C‐4′), 129.61 (C‐3′, C‐5′), 118.65 (C‐2′, C‐6′), 55.05 (C‐2), 26.46 (acetyl‐CH_3_) ppm. HRMS (ESI): calcd. for C_11_H_12_N_5_O_2_ [*M*+H]^+^ 246.0986, found 246.0984. TLC (CH_2_Cl_2_/MeOH 95 : 5): *R*_f_=0.40.

***N*** **‐(4‐Acetylphenyl)‐2‐(2*H*‐tetrazol‐5‐yl)acetamide (19)**: A suspension of nitrile **23** (102 mg, 0.505 mmol), NaN_3_ (159 mg, 2.45 mmol) and ZnBr_2_ (100 mg, 0.444 mmol) in *i*PrOH/water (1 : 3) was stirred under reflux for 2 d. After cooling to RT, EtOAc (200 mL) was added, and the organic layer was washed with HCl (0.2 m, 3×50 mL) and brine (50 mL) and then dried over Na_2_SO_4_. The solvent was evaporated under reduced pressure, and the resultant crude product was purified by centrifugal TLC (CH_2_Cl_2_/MeOH 100:0→90 : 10) to give **19** as a white solid (41 mg, 33 %). ^1^H NMR (500 MHz, [D_6_]DMSO): *δ*=16.26 (br, 1H, 2′′‐NH), 10.78 (s, 1H, NH(C=O)), 7.95 (d, *J*=8.8 Hz, 2H, 3′‐H, 5′‐H), 7.72 (d, *J*=8.8 Hz, 2H, 2′‐H, 6′‐H), 4.20 (s, 2H, 2‐H), 2.53 (s, 3H, acetyl‐CH_3_) ppm. ^13^C NMR (126 MHz, [D_6_]DMSO): *δ*=196.56 (acetyl‐C=O), 165.73 (C‐1), 150.82 (C‐5′′, HMBC), 142.95 (C‐1′), 132.11 (C‐4′), 129.60 (C‐3′, C‐5′), 118.52 (C‐2′, C‐6′), 31.80 (C‐2), 26.48 (acetyl‐CH_3_) ppm. MS (ESI): *m/z*=246.0 [*M*+H]^+^. HRMS (ESI): calcd. for C_11_H_12_N_5_O_2_ [*M*+H]^+^ 246.0986, found 246.0980. TLC (CH_2_Cl_2_/MeOH/HCOOH 94 : 5 : 1): *R*_f_=0.50.

***N*** **‐(4‐Acetylphenyl)‐2‐(1*H*‐imidazol‐4‐yl)acetamide (20)**: 2‐(1*H*‐imidazol‐4‐yl)acetic acid hydrochloride (180 mg, 1.11 mmol), HBTU (430 mg, 1.13 mmol) and DIPEA (140 μL, 0.823 mmol) were dissolved in DMF (5 mL). After 5 min stirring at RT, *p*‐aminoacetophenone (101 mg, 0.747 mmol) was added, and the reaction mixture was stirred at RT for 27 h. EtOAc (200 mL) was added, and the organic layer was washed with NaHCO_3_ solution (1.0 m, 3×50 mL) and brine (50 mL) and then dried over Na_2_SO_4_. The solvent was evaporated under reduced pressure, and the resultant crude product was purified by column chromatography (CH_2_Cl_2_/MeOH/NEt_3_ 90 : 5 : 5) to give **20** as a white solid (70 mg, 39 %). ^1^H NMR (500 MHz, [D_6_]DMSO): *δ*=11.95 (br, 1H, 1′′‐NH), 10.44 (s, 1H, NH(C=O)), 7.92 (d, *J*=8.8 Hz, 2H, 3′‐H, 5′‐H), 7.73 (d, *J*=8.8 Hz, 2H, 2′‐H, 6′‐H), 7.58 (d, *J*=1.0 Hz, 1H, imidazole‐H), 6.94 (s, 1H, imidazole‐H), 3.61 (s, 2H, 2‐H), 2.52 (s, 3H, acetyl‐CH_3_) ppm. ^13^C NMR (126 MHz, [D_6_]DMSO): *δ*=196.46 (acetyl‐C=O), 169.29 (C‐1), 143.57 (C‐1′), 134.95 (imidazole‐C), 131.60 (C‐4′), 129.47 (C‐3′, C‐5′), 118.25 (C‐2′, C‐6′), 35.87 (C‐2), 26.40 (acetyl‐CH_3_) ppm (due to poor relaxation, two imidazole‐carbon nuclei could not be observed, but the respective hydrogen nuclei were found in the ^1^H NMR spectrum). HRMS (ESI): calcd. for C_13_H_14_N_3_O_2_ [*M*+H]^+^ 244.1081, found 244.1077. TLC (CH_2_Cl_2_/MeOH/NEt_3_ 85 : 10 : 5): *R*_f_=0.25.

***N*** **‐(4‐Acetylphenyl)‐2‐chloroacetamide (21)**: *p*‐Aminoacetophenone (1.00 g, 7.41 mmol) and DIPEA (1.40 mL, 8.18 mmol) were dissolved in THF (5 mL). Chloroacetyl chloride (650 μL, 8.16 mmol) was added dropwise, and the reaction mixture was stirred at RT for 20 min. The reaction was quenched by addition of MeOH (15 mL). The mixture was then diluted with EtOAc (200 mL), washed with HCl (0.2 m, 3×30 mL) and sat. NaHCO_3_ solution (3×30 mL) and then dried over Na_2_SO_4_. The solvent was evaporated under reduced pressure to give **21** as a slightly green solid (1.54 g, 98 %). ^1^H NMR (500 MHz, CDCl_3_): *δ*=8.41 (s, 1H, NH), 7.98 (d, *J*=8.7 Hz, 2H, 3′‐H, 5′‐H), 7.68 (d, *J*=8.7 Hz, 2H, 2′‐H, 6′‐H), 4.22 (s, 2H, 2‐H), 2.59 (s, 3H, acetyl‐CH_3_) ppm. ^13^C NMR (126 MHz, CDCl_3_): *δ*=196.95 (acetyl‐C=O), 164.14 (C‐1), 141.00 (C‐1′), 133.90 (C‐4′), 129.89 (C‐3′, C‐5′), 119.39 (C‐2′, C‐6′), 42.99 (C‐2), 26.61 (acetyl‐CH_3_) ppm. HRMS (ESI): calcd. for C_10_H_11_ClNO_2_ [*M*+H]^+^ 212.0473, found 212.0466. TLC (CH_2_Cl_2_/MeOH 95 : 5): *R*_f_=0.55.

***N*** **‐(4‐Acetylphenyl)‐2‐iodoacetamide (22)**: Alkyl chloride **21** (364 mg, 1.72 mmol) and KI (859 mg, 5.18 mmol) were dissolved in acetone (20 mL), and the reaction mixture was stirred at RT for 15 h. EtOAc (200 mL) was added, the organic layer was washed with NaHCO_3_ solution (0.5 m, 3×50 mL), HCl (0.5 m, 3×50 mL) and brine (50 mL) and then dried over Na_2_SO_4_. The solvent was evaporated under reduced pressure to give **22** as a slightly orange solid (472 mg, 91 %). ^1^H NMR (500 MHz, CDCl_3_+CD_3_OD): *δ*=7.91 (d, *J*=9.0 Hz, 2H, 3′‐H, 5′‐H), 7.63 (d, *J*=8.8 Hz, 2H, 2′‐H, 6′‐H), 3.83 (s, 2H, 2‐H), 2.55 (s, 3H, acetyl‐CH_3_) ppm. ^13^C NMR (126 MHz, CDCl_3_+CD_3_OD): *δ*=197.53 (acetyl‐C=O), 166.56 (C‐1), 142.42 (C‐1′), 133.09 (C‐4′), 129.78 (C‐3′, C‐5′), 119.04 (C‐2′, C‐6′), 26.50 (acetyl‐CH_3_), −0.71 (C‐2) ppm. HRMS (ESI): calcd. for C_10_H_11_INO_2_ [*M*+H]^+^ 303.9829, found 303.9824. TLC (petroleum ether/EtOAc 7 : 3): *R*_f_=0.15.

***N*** **‐(4‐Acetylphenyl)‐2‐cyanoacetamide (23)**: Cyanoacetic acid (141 mg, 1.66 mmol), DIPEA (580 μL, 3.33 mmol) and EDC hydrochloride (428 mg, 2.23 mmol) were dissolved in CH_2_Cl_2_ (10 mL) and the mixture was stirred at RT for 15 min. Then, *p*‐aminoacetophenone (205 mg, 1.52 mmol) was added, and the reaction mixture was stirred at RT for 22 h. EtOAc (250 mL) was added, the organic layer was washed with water (2×50 mL), HCl (0.2 m, 1×50 mL) and brine (50 mL) and then dried over Na_2_SO_4_. The solvent was evaporated under reduced pressure, and the resultant crude product was purified by column chromatography (CH_2_Cl_2_/MeOH 98 : 2) to give **23** as a white solid (176 mg, 57 %). ^1^H NMR (500 MHz, [D_6_]DMSO): *δ*=10.62 (s, 1H, NH), 7.95 (d, *J*=8.8 Hz, 2H, 3′‐H, 5′‐H), 7.68 (d, *J*=8.8 Hz, 2H, 2′‐H, 6′‐H), 3.96 (s, 2H, 2‐H), 2.53 (s, 3H, acetyl‐CH_3_) ppm. ^13^C NMR (126 MHz, [D_6_]DMSO): *δ*=196.53 (acetyl‐C=O), 161.71 (C‐1), 142.60 (C‐1′), 132.25 (C‐4′), 129.58 (C‐3′, C‐5′), 118.52 (C‐2′, C‐6′), 115.69 (CN), 27.01 (C‐2), 26.46 (acetyl‐CH_3_) ppm. HRMS (ESI): calcd. for C_11_H_11_N_2_O_2_ [*M*+H]^+^ 203.0815, found 203.0813. TLC (CH_2_Cl_2_/MeOH 95 : 5): *R*_f_=0.35.

**Sodium 2‐((4‐acetylphenyl)amino)‐2‐oxoethane‐1‐sulfonate (24)**: To a suspension of alkyl chloride **21** (509 mg, 2.40 mmol) in EtOH (6 mL), a solution of Na_2_SO_3_ (302 mg, 2.40 mmol) in water (6 mL) was added. The reaction mixture was stirred under reflux for 4 h. The formed precipitate was filtered off, washed with cold water and dried to give **24** as a white solid (473 mg, 70 %). ^1^H NMR (500 MHz, [D_6_]DMSO): *δ*=10.31 (s, 1H, NH), 7.92 (d, *J*=8.8 Hz, 2H, 3′‐H, 5′‐H), 7.71 (d, *J*=8.8 Hz, 2H, 2′‐H, 6′‐H), 3.57 (s, 2H, 1‐H), 2.52 (s, 3H, acetyl‐CH_3_) ppm. ^13^C NMR (126 MHz, [D_6_]DMSO): *δ*=196.49 (acetyl‐C=O), 165.01 (C‐2), 143.43 (C‐1′), 131.58 (C‐4′), 129.47 (C‐3′, C‐5′), 118.18 (C‐2′, C‐6′), 59.10 (C‐1), 26.42 (acetyl‐CH_3_) ppm. HRMS (ESI): calcd. for C_10_H_12_NO_5_S [*M*(acid)+H]^+^ 258.0431, found 258.0428. TLC (CH_2_Cl_2_/MeOH 95 : 5): *R*_f_=0.00.

**Sodium (2‐((4‐acetylphenyl)amino)‐2‐oxoethyl)(methyl)phosphinate (25)**: General procedure **GP2** with ethyl ester **27** (50 mg, 0.18 mmol) to give **25** as a white solid (23 mg, 44 %). ^1^H NMR (500 MHz, [D_6_]DMSO): *δ*=10.77 (s, 1H, NH), 7.87 (d, *J*=8.8 Hz, 2H, 3′‐H, 5′‐H), 7.71 (d, *J*=8.8 Hz, 2H, 2′‐H, 6′‐H), 2.96 (d, *J*=17.7 Hz, 2H, 1‐H), 2.50 (br, 3H (under solvent signal), acetyl‐CH_3_), 1.42 (d, *J*=14.8 Hz, 3H, PCH_3_) ppm. ^13^C NMR (126 MHz, [D_6_]DMSO): *δ*=196.42 (acetyl‐C=O), 166.00 (C‐1), 143.69 (C‐1′), 131.51 (C‐4′), 129.37 (C‐3′, C‐5′), 118.17 (C‐2′, C‐6′), 42.13 (d, *J*_CP_=78.7 Hz, C‐1), 26.36 (acetyl‐CH_3_), 16.29 (d, *J*_CP_=97.7 Hz, PCH_3_) ppm. ^31^P NMR (162 MHz, [D_6_]DMSO): *δ*=37.80 ppm. HRMS (ESI): calcd. for C_11_H_15_NO_4_P [*M*(acid)+H]^+^ 256.0733, found: 256.0730. TLC (CH_2_Cl_2_/MeOH/HCOOH 85 : 13 : 2): *R*_f_=0.05.

**Disodium (2‐((4‐acetylphenyl)amino)‐2‐oxoethyl)phosphonate (26)**: General procedure **GP2** with ethyl ester **28** (182 mg, 0.581 mmol) to give **26** as a slightly orange solid (89 mg, 60 %). ^1^H NMR (500 MHz, CD_3_OD): *δ*=8.62 (d, *J*=8.8 Hz, 3′‐H, 5′‐H), 8.27 (d, *J*=8.8 Hz, 2′‐H, 6′‐H), 3.64 (d, *J*=21.0 Hz, 1‐H), 3.26 (s, 3H, acetyl‐CH_3_) ppm. ^13^C NMR (126 MHz, CD_3_OD): *δ*=213.57 (acetyl‐C=O), 179.12 (C‐2), 153.54 (C‐1′), 143.67 (C‐4′), 141.13 (C‐3′, C‐5′), 131.27 (C‐2′, C‐6′), 49.60 (d, *J*_CP_=122.2 Hz, C‐1), 37.16 (acetyl‐CH_3_). ^31^P NMR (203 MHz, CD_3_OD): *δ*=15.82 ppm. HRMS (ESI): calcd. for C_13_H_21_NO_4_P [*M*(acid)+H]^+^ 258.0526, found: 258.0523.

**Ethyl (2‐((4‐acetylphenyl)amino)‐2‐oxoethyl)(methyl)phosphinate (27)**: Alkyl chloride **21** (113 mg, 0.536 mmol) was dissolved in a mixture of toluene (2 mL) and THF (2 mL). At 100 °C, diethyl methylphosphonate (200 μL, 1.33 mmol) was added dropwise over 10 min. The reaction mixture was stirred at 100 °C for 23 h. After cooling to RT, the solvent was evaporated under reduced pressure, and the resultant crude product was purified by column chromatography (CH_2_Cl_2_/MeOH 98 : 2) to give **27** as a white solid (113 mg, 75 %). ^1^H NMR (500 MHz, CDCl_3_): *δ*=9.97 (s, 1H, NH), 7.73 (d, *J*=8.8 Hz, 2H, 3′‐H, 5′‐H), 7.51 (d, *J*=8.8 Hz, 2H, 2′‐H, 6′‐H), 4.19–4.12 (m, 2H, ethyl‐1‐H), 3.19 (dd, *J*=18.9, 14.3 Hz, 1H, 1‐H_a_), 2.99 (dd, *J*=14.3, 14.3 Hz, 1H, 1‐H_b_), 2.50 (s, 3H, acetyl‐CH_3_), 1.72 (d, *J*=14.5 Hz, 3H, PCH_3_), 1.37 (t, *J*=7.0 Hz, 3H, ethyl‐2‐H) ppm. ^13^C NMR (126 MHz, CDCl_3_): *δ*=197.02 (acetyl‐C=O), 163.42 (d, *J*_CP_=3.8 Hz, C‐2), 142.54 (C‐1′), 132.74 (C‐4′), 129.52 (C‐3′, C‐5′), 118.86 (C‐2′, C‐6′), 61.57 (d, *J*_CP_=6.5 Hz, ethyl‐C‐1), 40.35 (d, *J*_CP_=81.2 Hz, C‐1), 26.48 (acetyl‐CH_3_), 16.71 (d, *J*_CP_=6.2 Hz, ethyl‐C‐2), 14.78 (d, *J*_CP_=97.3 Hz, PCH_3_) ppm. ^31^P NMR (203 MHz, CDCl_3_): *δ*=49.31 ppm. HRMS (ESI): calcd. for C_13_H_18_NO_4_P [*M*+H]^+^ 284.1046, found 284.1043. TLC (CH_2_Cl_2_/MeOH 95 : 5): *R*_f_=0.15.

**Diethyl (2‐((4‐acetylphenyl)amino)‐2‐oxoethyl)phosphonate (28)**: Alkyl chloride **21** (146 mg, 0.692 mmol) was suspended in triethylphosphite (2.30 mL, 13.3 mmol) and the reaction mixture was stirred under reflux for 18 h. After cooling to RT, the solvent was evaporated under reduced pressure, and the resultant oily crude product was purified by column chromatography (petroleum ether/EtOAc 1 : 2→1 : 5) to give **28** as a white solid (193 mg, 89 %). ^1^H NMR (500 MHz, CDCl_3_): *δ*=9.53 (s, 1H, NH), 7.81 (d, *J*=8.7 Hz, 2H, 3′‐H, 5′‐H), 7.56 (d, *J*=8.8 Hz, 2H, 2′‐H, 6′‐H), 4.24–4.18 (m, 4H, ethyl‐1‐H), 3.08 (d, *J*=21.1 Hz, 2H, 1‐H), 2.53 (s, 3H, acetyl‐CH_3_), 1.38 (t, *J*=7.1 Hz, 6H, ethyl‐2‐H) ppm. ^13^C NMR (126 MHz, CDCl_3_): *δ*=197.03 (acetyl‐C=O), 162.61 (d, *J*=3.9 Hz, C‐2), 142.32 (C‐1′), 132.94 (C‐4′), 129.65 (C‐3′, C‐5′), 118.98 (C‐2′, C‐6′), 63.33 (d, *J*_CP_=6.8 Hz, ethyl‐C‐1), 36.50 (d, *J*_CP_=129.3 Hz, C‐1), 26.51 (acetyl‐CH_3_), 16.49 (d, *J*_CP_=6.1 Hz, ethyl‐C‐2) ppm. ^31^P NMR (203 MHz, CDCl_3_): *δ*=22.49 ppm. MS (ESI): *m/z*=336.0 [*M*+Na]^+^. TLC (CH_2_Cl_2_/MeOH 9 : 1): *R*_f_=0.55.

**FRET‐based collagenase inhibition assay**: The peptidase domain (PD) of ColH (Uniprot: Q46085; Leu331‐Gly721) and the collagenase unit of ColQ1 (ColQ1‐CU; Uniprot: B9 J3S4; Tyr94‐Gly765) were expressed and purified as previously described.^[24]^ IC_50_ measurements were performed as previously reported.^[13]^ ColH‐PD was pretreated with the compounds at RT for 1 h. The reaction was initiated by the addition of 2 μm of the peptide substrate Mca‐Ala‐Gly‐Pro‐Pro‐Gly‐Pro‐Dpa‐Gly‐Arg‐NH_2_ (FS1‐1; Mca=(7‐methoxycoumarin‐4‐yl)acetyl; Dpa=*N*‐3‐(2,4‐dinitrophenyl)‐l‐2,3‐diaminopropionyl). The increase in fluorescence was monitored for 2 min (*λ*_ex_=328 nm; *λ*_em_=392 nm) at 25 °C. The final concentrations were 10 nm ColH‐PD and 1 nM ColQ1‐CU, respectively, 2 μm FS1‐1, 250 mm HEPES pH 7.5, 400 mm NaCl, 10 mm CaCl_2_, 10 μm ZnCl_2_, 2 % DMSO and eight different compound concentrations. The percentage of enzyme inhibition was calculated in relation to a blank reference without compound added. All experiments were performed in triplicates and repeated at least three times. IC_50_ values were determined using nonlinear regression with a constant Hill slope of −1. Regression analysis was performed using GraphPad Prism 5 (Graph Pad Software, San Diego, CA, USA).

**MMP inhibition assay**: The catalytic domains of MMP‐1, −2, −3, −7, −8, and −14 along with the SensoLyte 520 generic MMP activity kit were purchased from AnaSpec (Fremont, CA, USA). The assay was performed as previously described using Batimastat as a positive control[^13^, ^25^] and according to the guidelines of the manufacturer.

**HDAC inhibition assay**: HDAC3 and HDAC8 inhibitor screening kits were purchased from Sigma–Aldrich. The assay was performed according to the guidelines of the manufacturer. Fluorescence signals were measured in a CLARIOstar plate reader (BMG Labtech).

**TACE inhibition assay**: A TACE (ADAM‐17) inhibitor screening assay kit was purchased from Sigma–Aldrich. The assay was performed according to the guidelines of the manufacturer. Fluorescence signals were measured in a CLARIOstar plate reader (BMG Labtech).

**Cytotoxicity assay**: HepG2, HEK293, or A549 cells (2×10^5^ cells per well) were seeded in 24‐well, flat‐bottomed plates. Culturing of cells, incubations, and OD measurements were performed as previously described^[26]^ with minor modifications. 24 h after seeding the cells, the incubation was started by the addition of compounds at a final DMSO concentration of 1 %. The living cell mass was determined after 48 h. At least two independent measurements were performed for each compound.

**Zebrafish embryo toxicity assay**: Toxicity testing was performed according to the procedure described in the literature^[27]^ with minor modifications using zebrafish embryos of the AB wild‐type line at 1 d post fertilisation (dpf) as previously reported.^[16]^ All of the described experiments were performed with zebrafish embryos <120 h post‐fertilisation (hpf) and are not classified as animal experiments according to EU Directive 2010/63/EU. Protocols for husbandry and care of adult animals were in accordance with the German Animal Welfare Act (§11 Abs. 1 TierSchG).

***Ex vivo*** **pig skin degradation assay**: The assay was performed as previously reported^[16]^ using explants from pig ears in a 24‐well plate. Compound **26** was preincubated with 300 nM of ColQ1, 4 mM CaCl_2_, 10 μM ZnCl_2_ in DMEM medium at 37 °C and 5 % CO_2_ for 1 h. After preincubation, one skin explant was added into each well and incubated at 37 °C and 5 % CO_2_ for 24 h under 300 rpm shaking. Hydroxyproline release was measured using a hydroxyproline assay kit (Sigma–Aldrich) according to the guidelines of the manufacturer. Absorbance was measured using a PHERAstar plate reader (BMG Labtech). The absorbance values were converted into hydroxyproline concentrations (μg/mL) using a calibration curve of hydroxyproline (Figures S1 and S2 in the Supporting Information). The absolute concentrations were converted into percentages by setting the concentration of the 0 μM value to 100 % and calculating all values from each experiment separately.

## Conflict of interest

The authors declare no conflict of interest.

## Supporting information

As a service to our authors and readers, this journal provides supporting information supplied by the authors. Such materials are peer reviewed and may be re‐organized for online delivery, but are not copy‐edited or typeset. Technical support issues arising from supporting information (other than missing files) should be addressed to the authors.

Supplementary
