# Supplementary material for: Phosphonate as a Stable Zinc‐Binding Group for “Pathoblocker” Inhibitors of Clostridial Collagenase H (ColH)
Source: ChemMedChem. 2021 Mar 16;16(8):1257–67. doi: 10.1002/cmdc.202000994 (PMC8251769; doi:10.1002/cmdc.202000994)
Supplement: Supplementary file 1 — Supplementary [file CMDC-16-1257-s001.pdf]

ChemMedChem

Supporting Information

# Phosphonate as a Stable Zinc-Binding Group for "Pathoblocker" Inhibitors of Clostridial Collagenase H (ColH)

Katrin Voos, Esther Schönauer, Alaa Alhayek, Jörg Haupenthal, Anastasia Andreas, Rolf Müller, Rolf W. Hartmann, Hans Brandstetter, Anna K. H. Hirsch,* and Christian Ducho*

**Table of Contents**

Additional data for the *ex vivo* pig skin degradation model…………………….................... S2

$^{1}$H, $^{13}$C and $^{31}$P NMR spectra of synthesised compounds................................................ S3

**Additional data for the *ex vivo* pig skin degradation model**

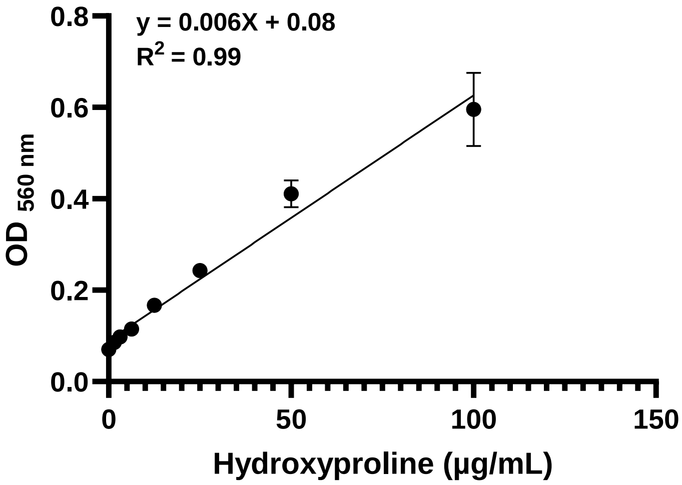


**Figure S1.** Calibration curve for hydroxyproline. Mean ± SD of three independent measurements are depicted.

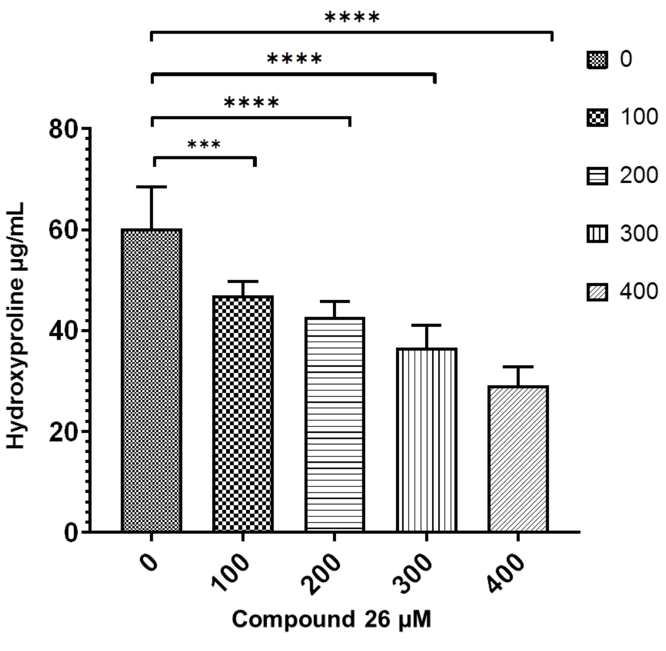


**Figure S2.** Amount of hydroxyproline release at different concentrations of **26**. Data shown represent the means ± SD from three independent measurements. One-way ANOVA followed by Tukey's HSD test (***: $p = 0.0003$; ****: $p < 0.0001$).

**$^{1}$H, $^{13}$C and $^{31}$P NMR spectra of synthesised compounds**

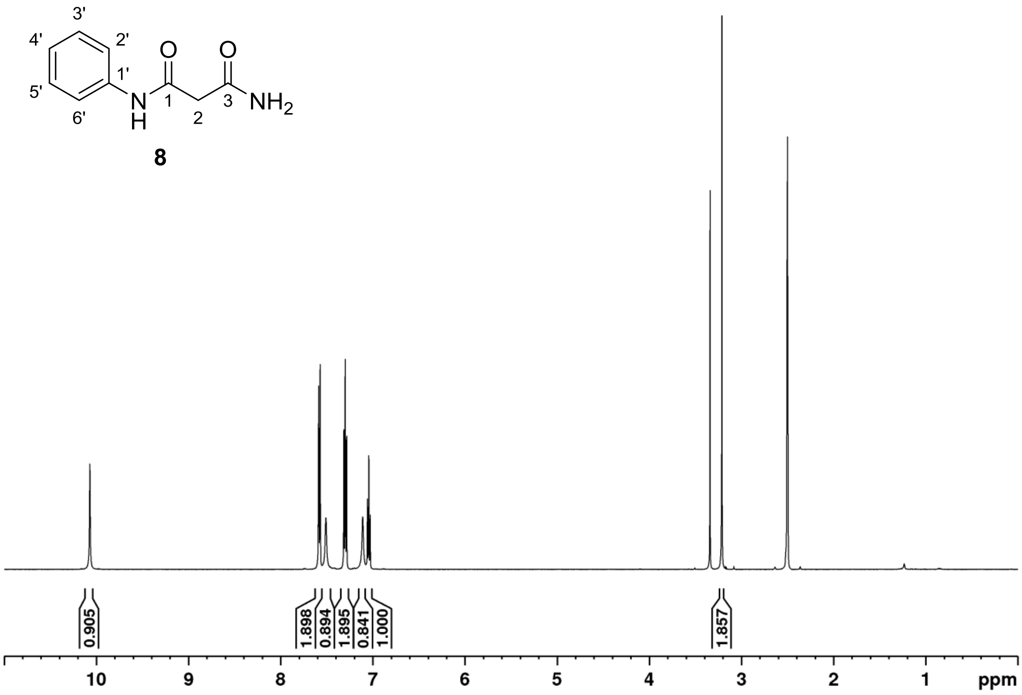


$^{1}$H NMR spectrum of **8** (500 MHz, DMSO-$d_6$).

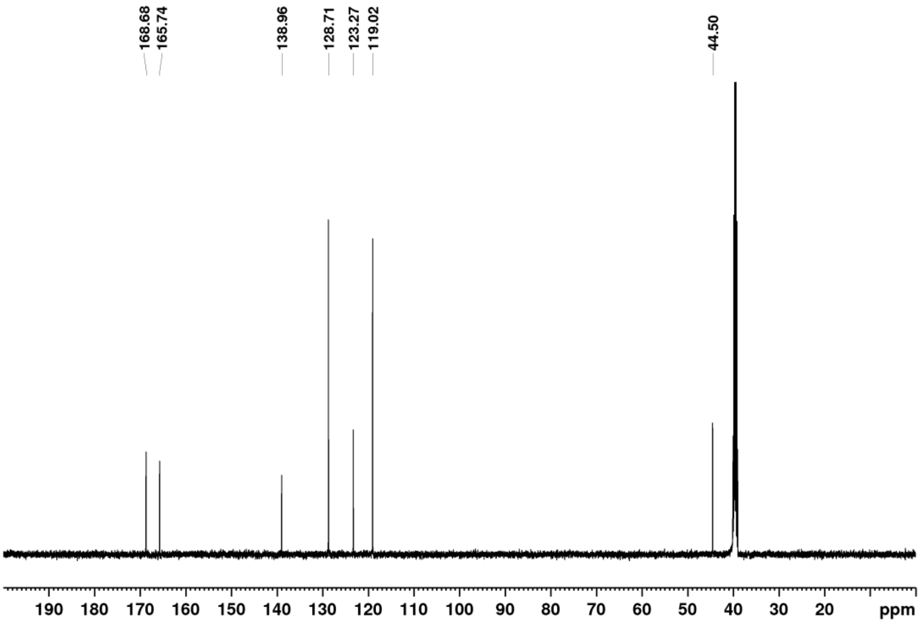


$^{13}$C NMR spectrum of **8** (126 MHz, DMSO-$d_6$).

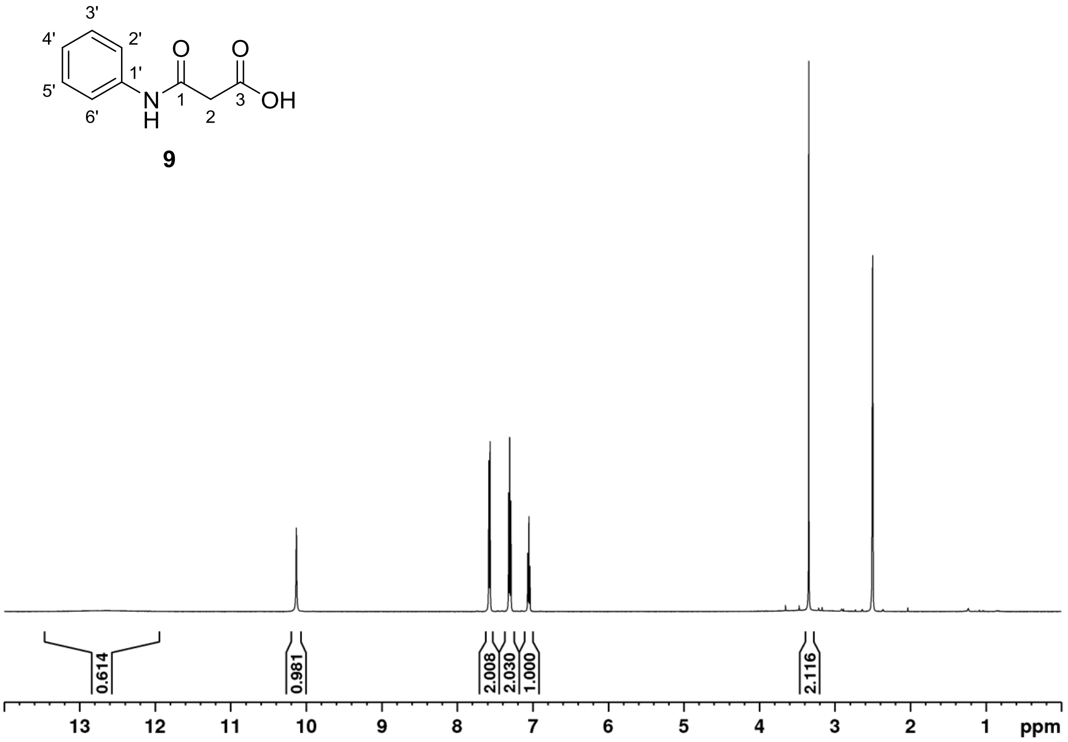


$^{1}$H NMR spectrum of **9** (500 MHz, DMSO-d$_6$).

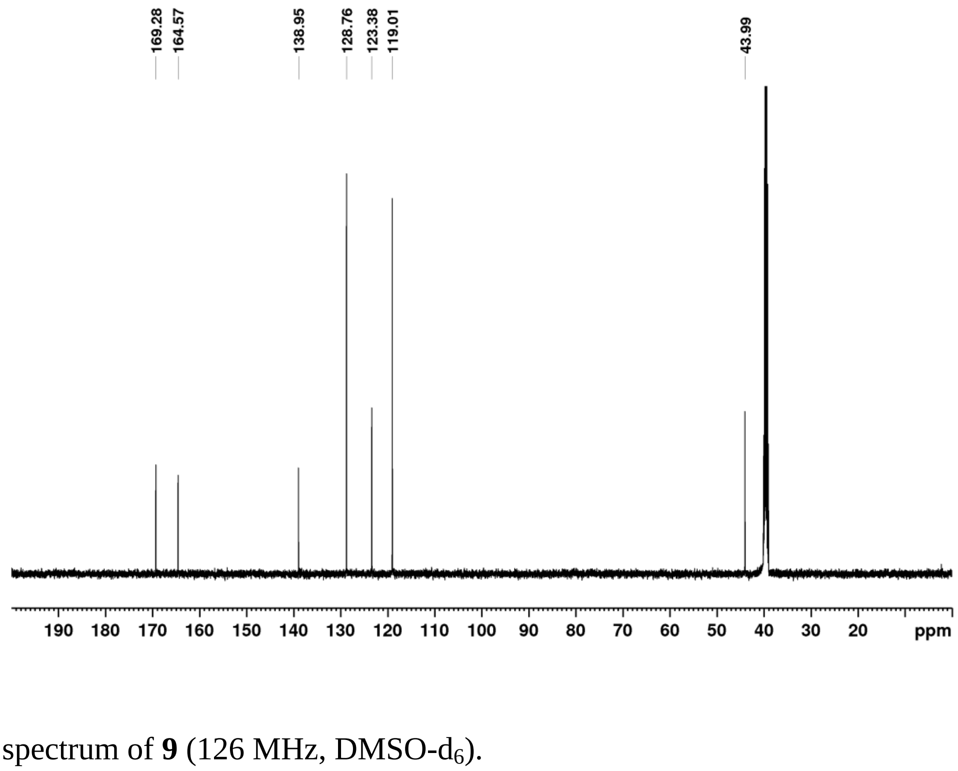


$^{13}$C NMR spectrum of **9** (126 MHz, DMSO-d$_6$).

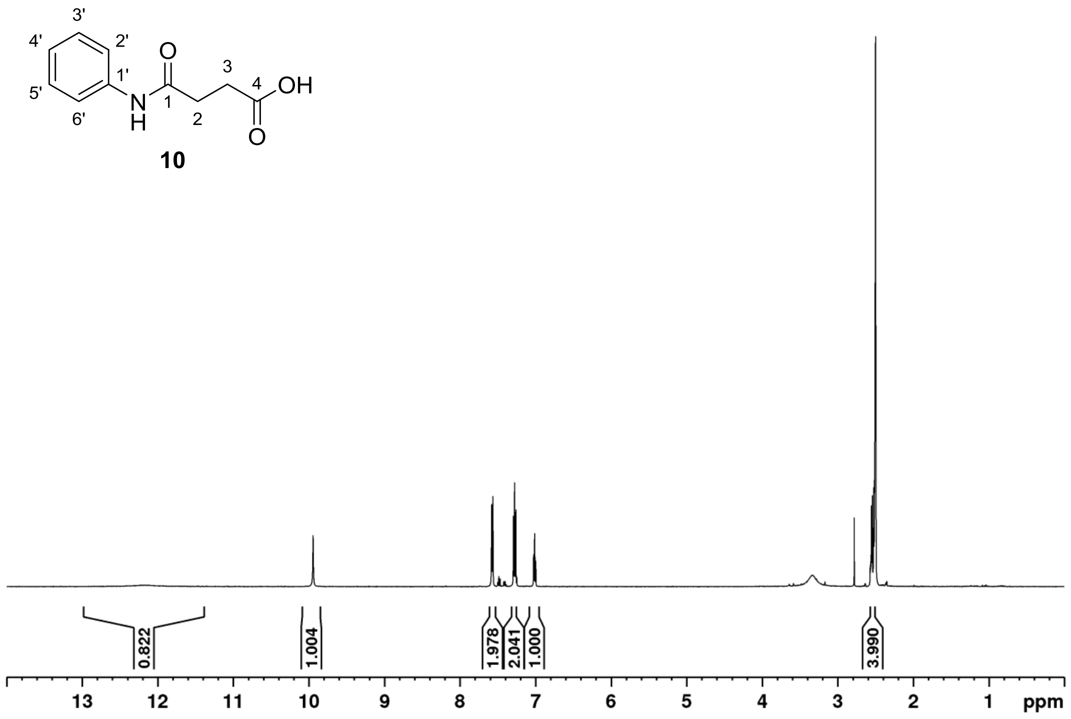


$^{1}$H NMR spectrum of **10** (500 MHz, DMSO-$d_6$).

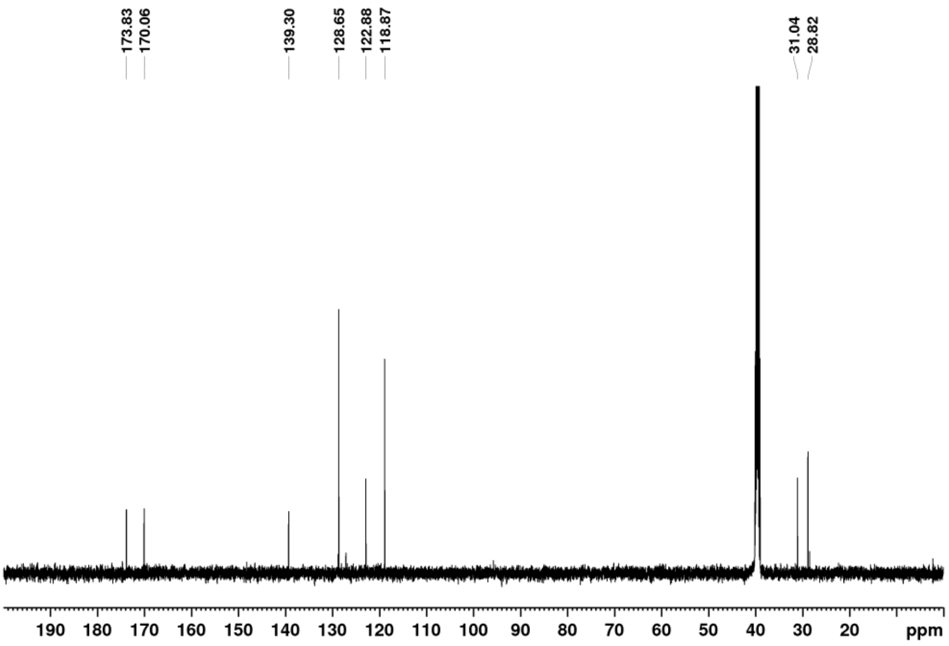


$^{13}$C NMR spectrum of **10** (126 MHz, DMSO-$d_6$).

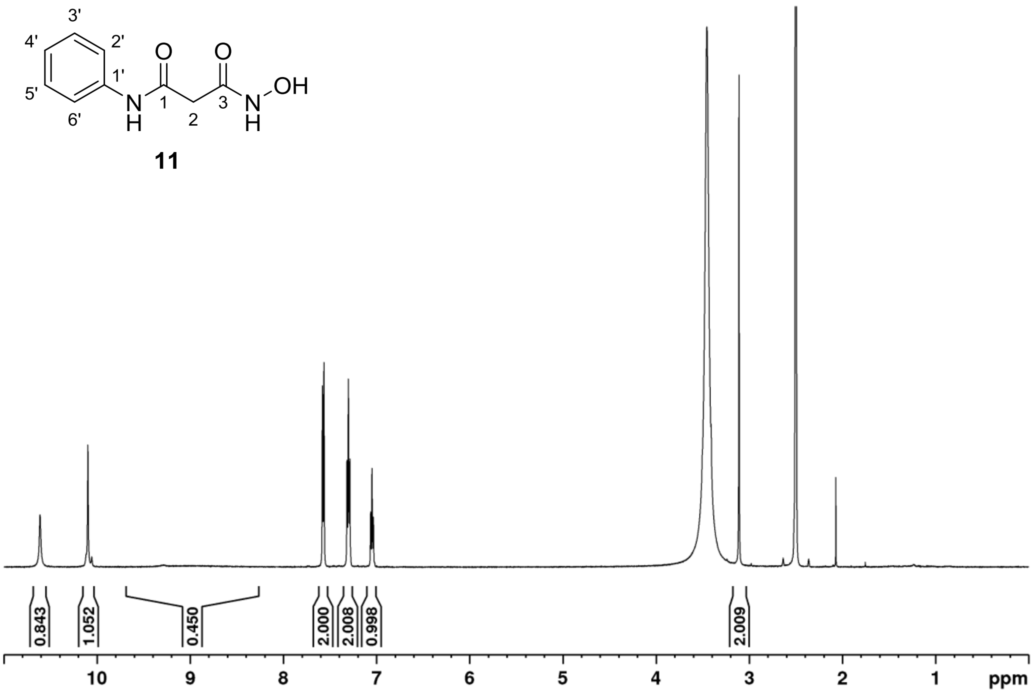


$^{1}H$ NMR spectrum of **11** (500 MHz, DMSO-$d_6$).

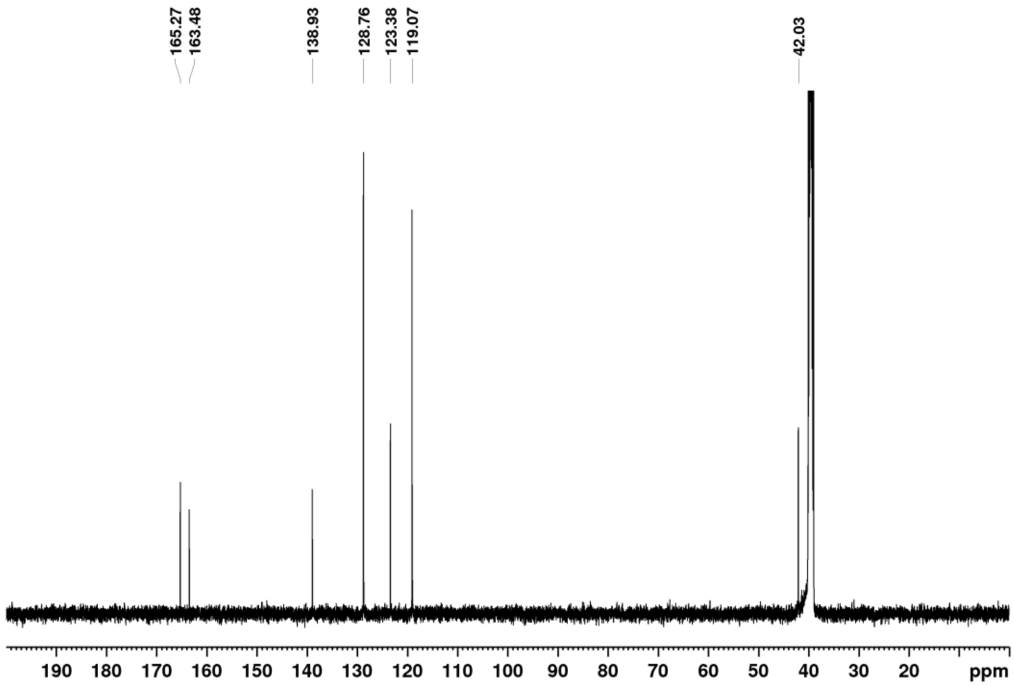


$^{13}C$ NMR spectrum of **11** (126 MHz, DMSO-$d_6$).

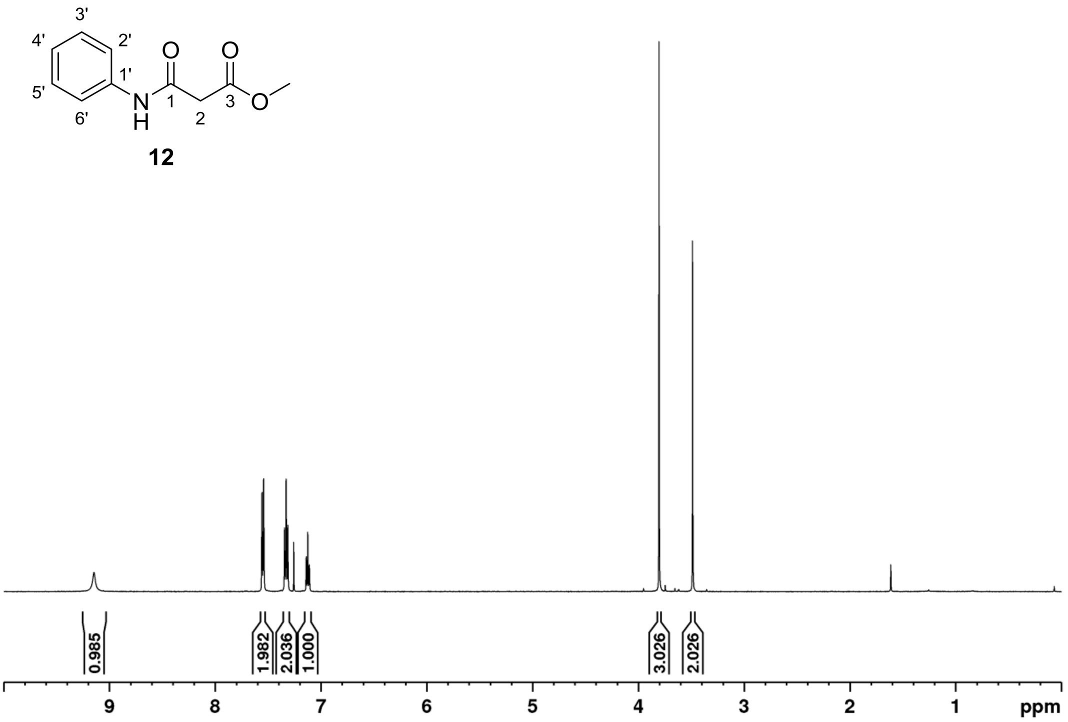


$^{1}H$ NMR spectrum of **12** (500 MHz, $CDCl_3$).

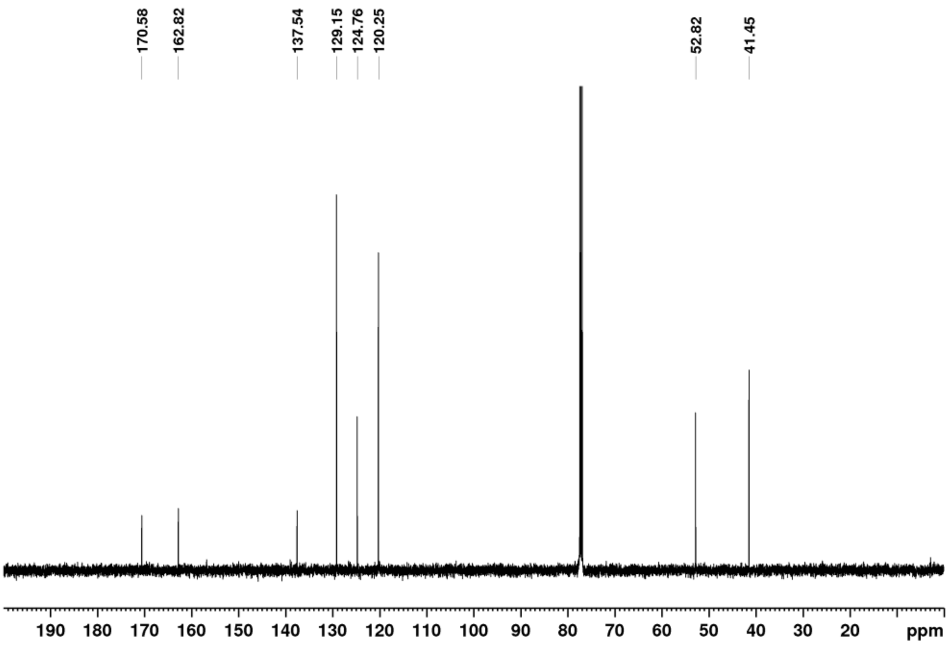


$^{13}C$ NMR spectrum of **12** (126 MHz, $CDCl_3$).

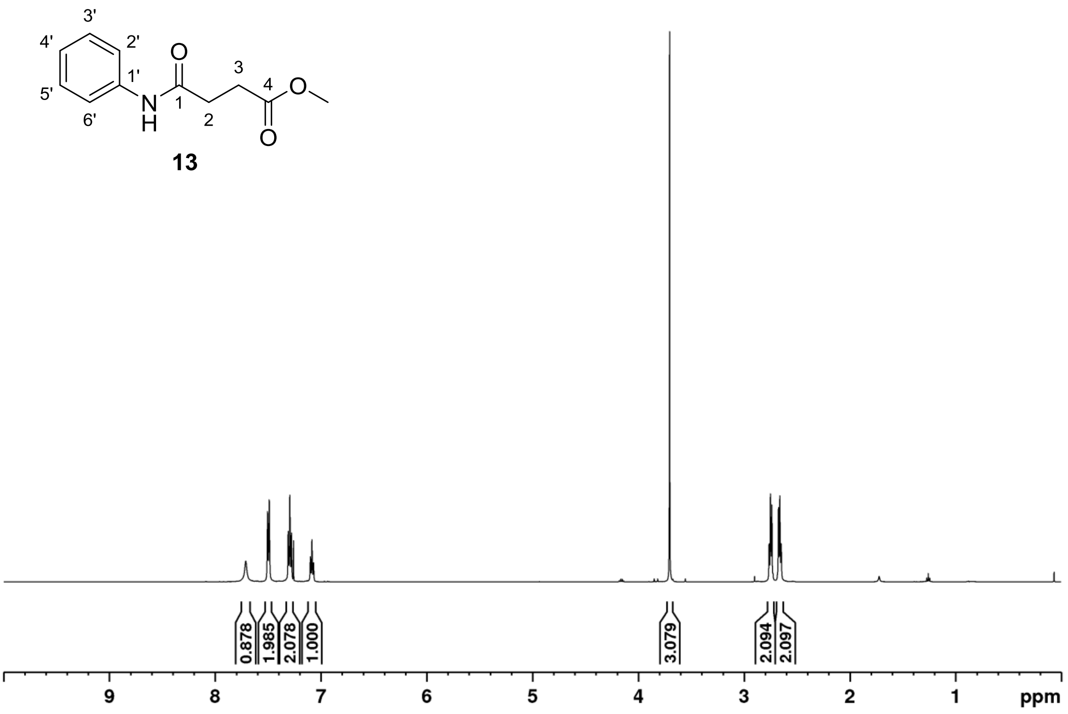


$^{1}$H NMR spectrum of **13** (500 MHz, $CDCl_3$).

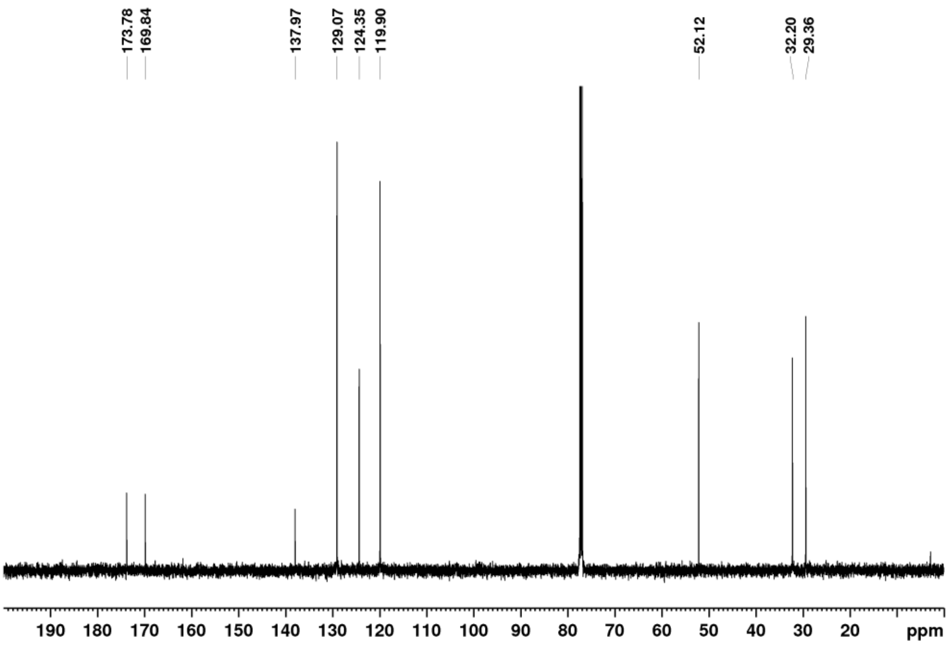


$^{13}$C NMR spectrum of **13** (126 MHz, $CDCl_3$).

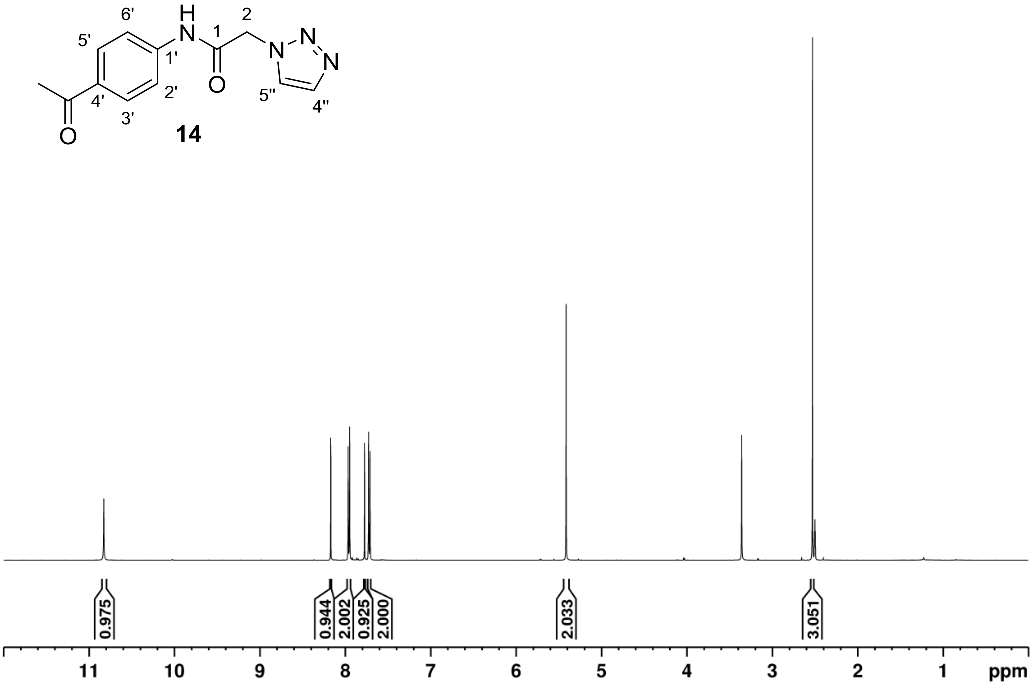


$^{1}$H NMR spectrum of **14** (500 MHz, DMSO-d$_6$).

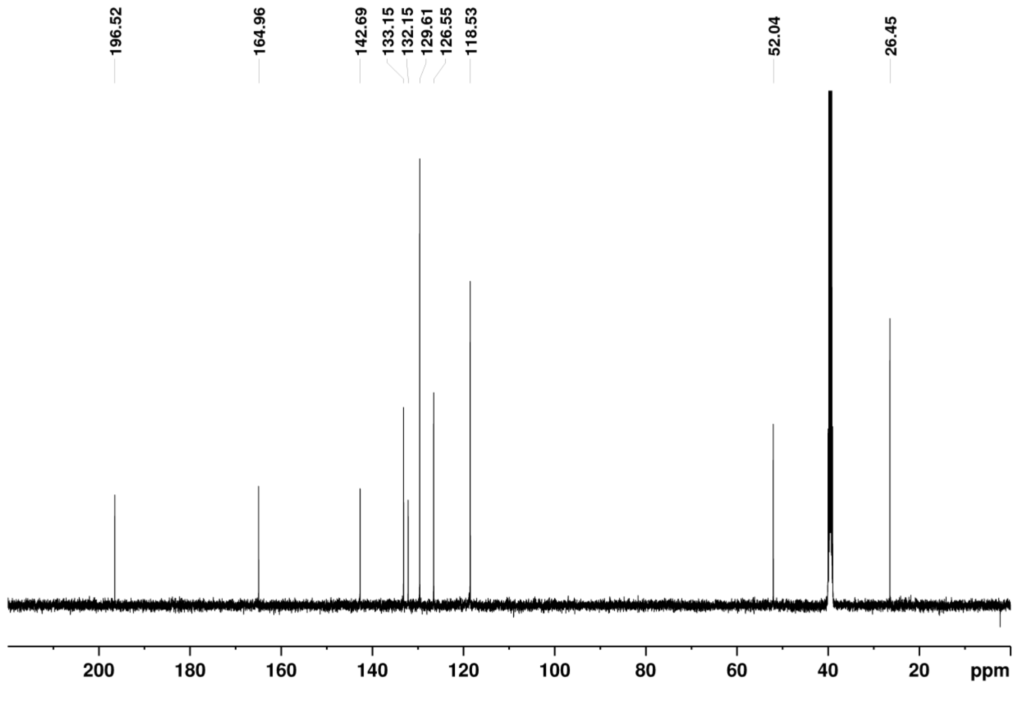


$^{13}$C NMR spectrum of **14** (126 MHz, DMSO-d$_6$).

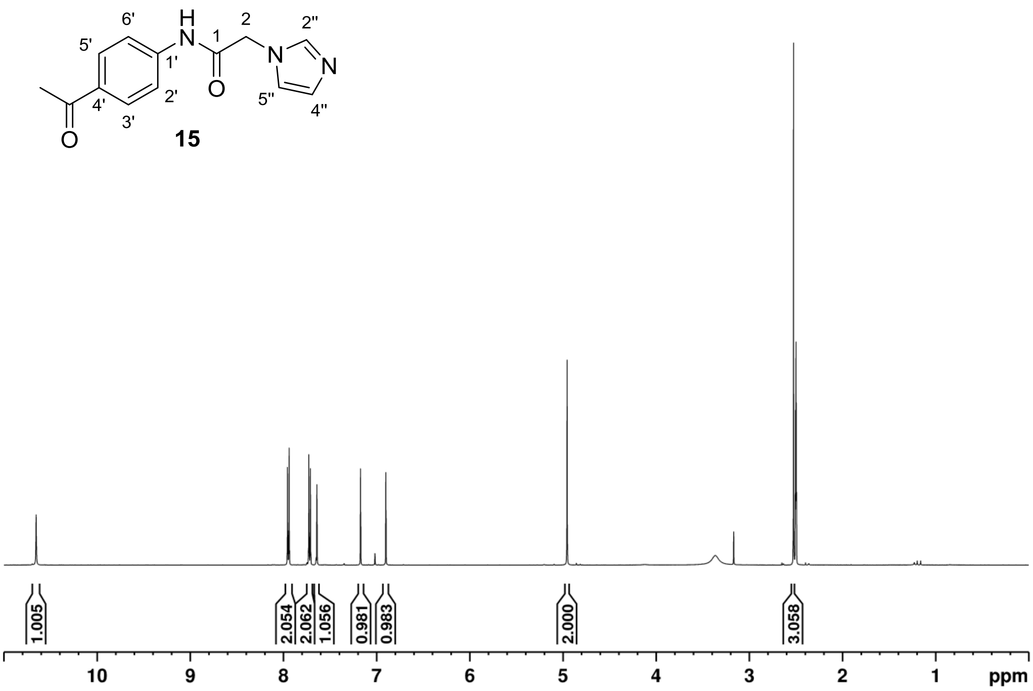


$^{1}$H NMR spectrum of **15** (500 MHz, DMSO-$d_6$).

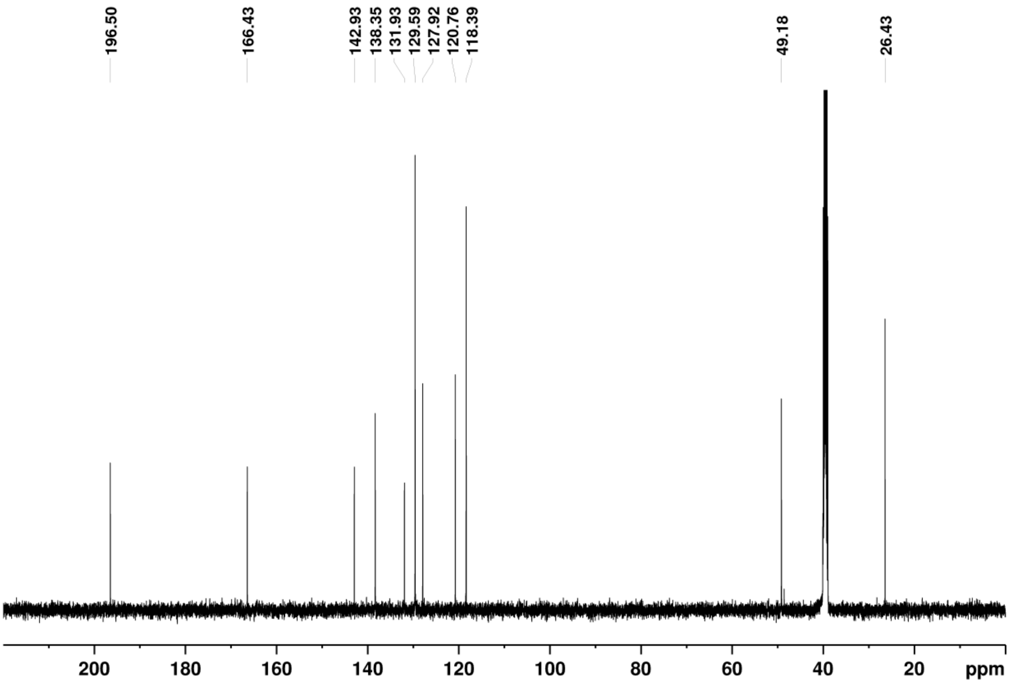


$^{13}$C NMR spectrum of **15** (126 MHz, DMSO-$d_6$).

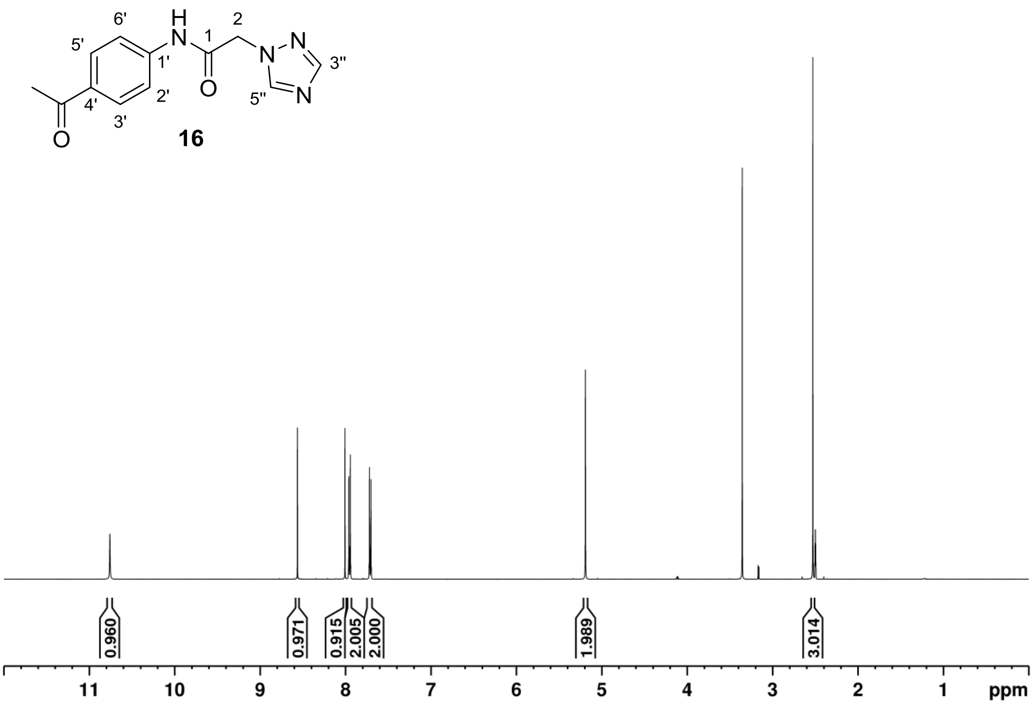


$^{1}$H NMR spectrum of **16** (500 MHz, DMSO-$d_6$).

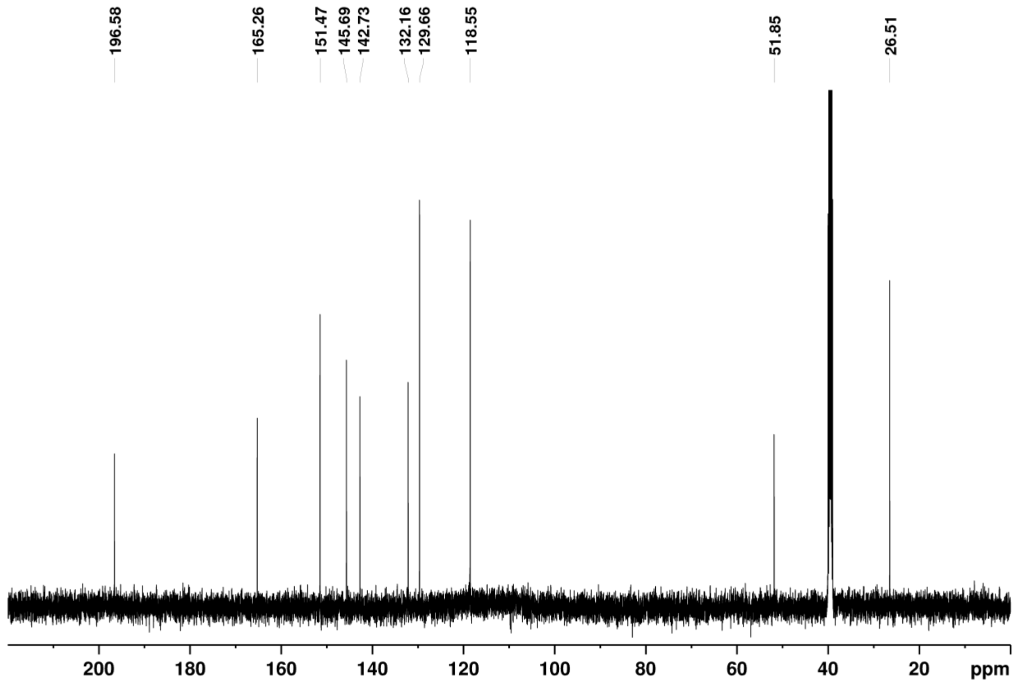


$^{13}$C NMR spectrum of **16** (126 MHz, DMSO-$d_6$).

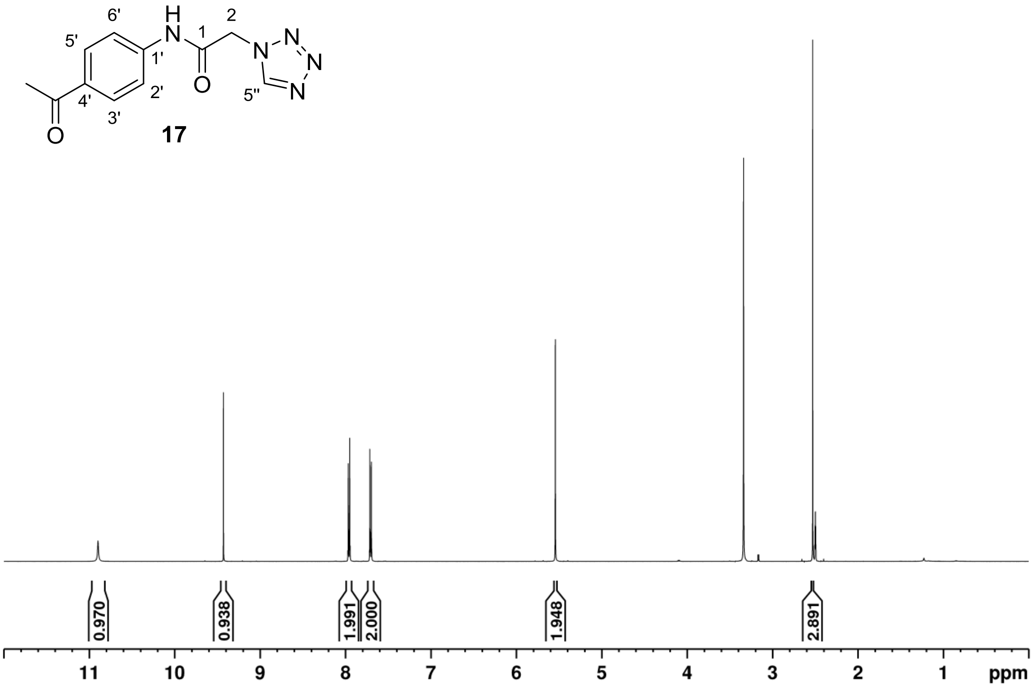


$^{1}$H NMR spectrum of **17** (500 MHz, DMSO-d$_6$).

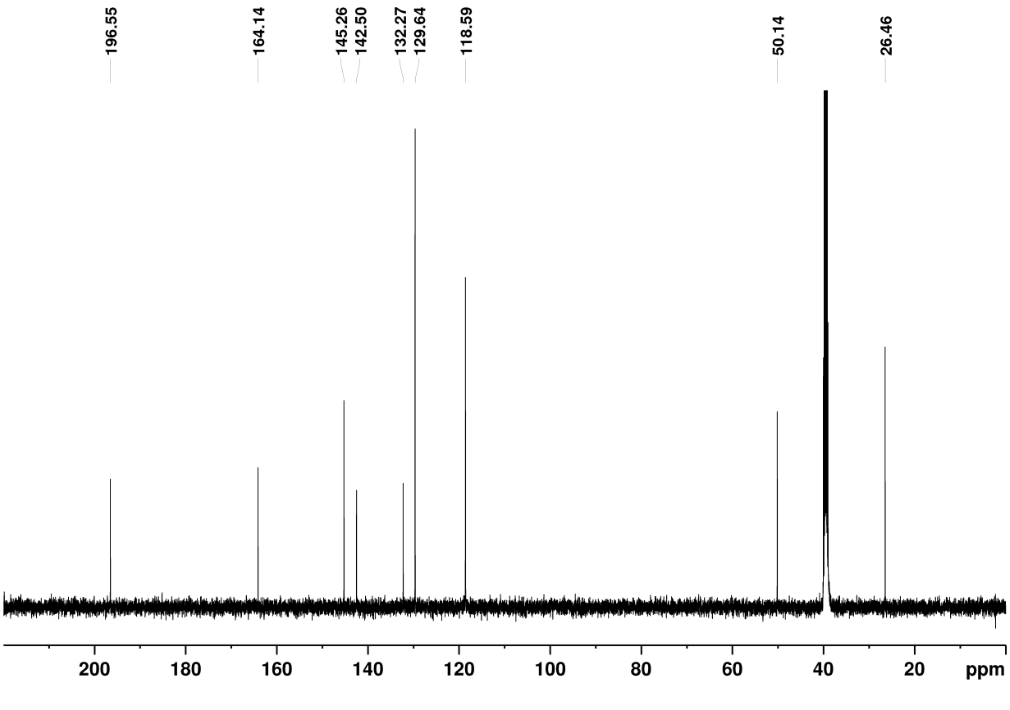


$^{13}$C NMR spectrum of **17** (126 MHz, DMSO-d$_6$).

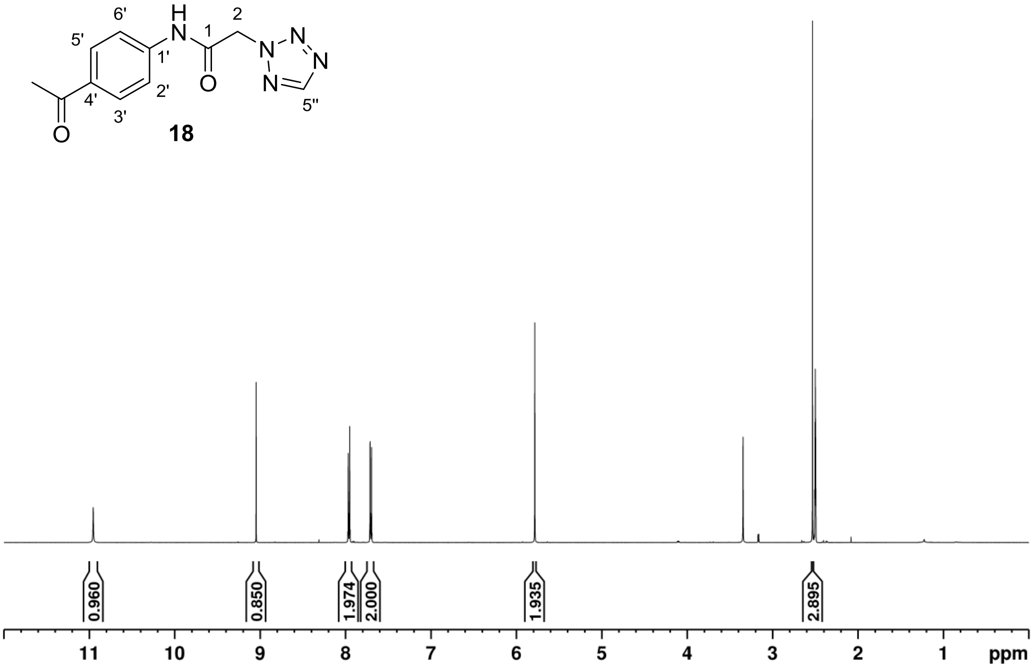


$^{1}$H NMR spectrum of **18** (500 MHz, DMSO-$d_6$).

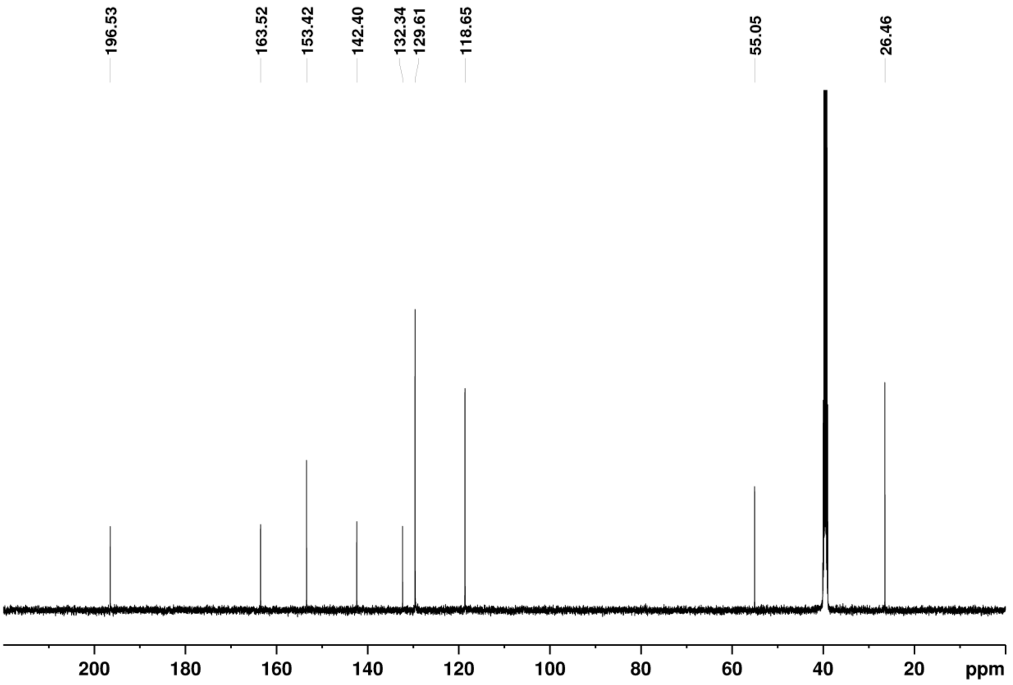


$^{13}$C NMR spectrum of **18** (126 MHz, DMSO-$d_6$).

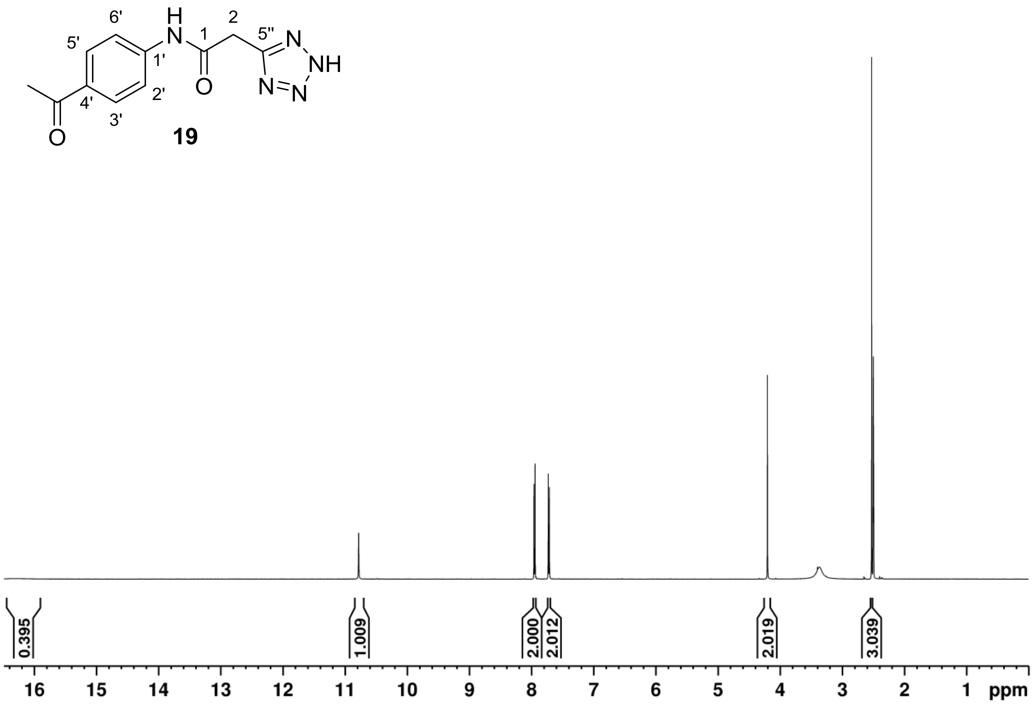


$^{1}$H NMR spectrum of **19** (500 MHz, DMSO-d$_6$).

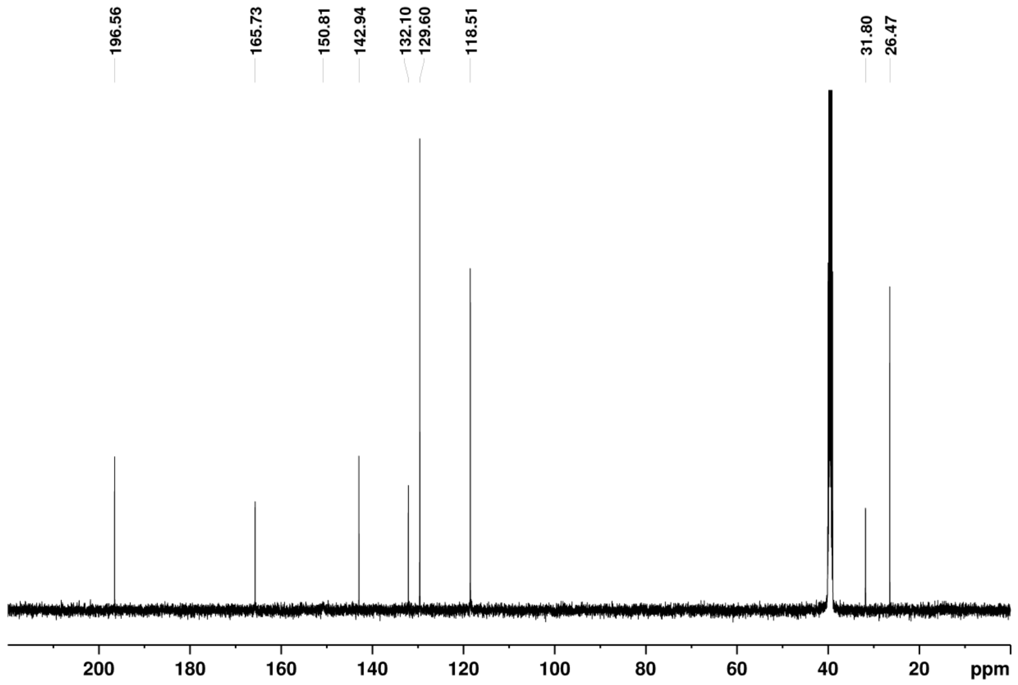


$^{13}$C NMR spectrum of **19** (126 MHz, DMSO-d$_6$).

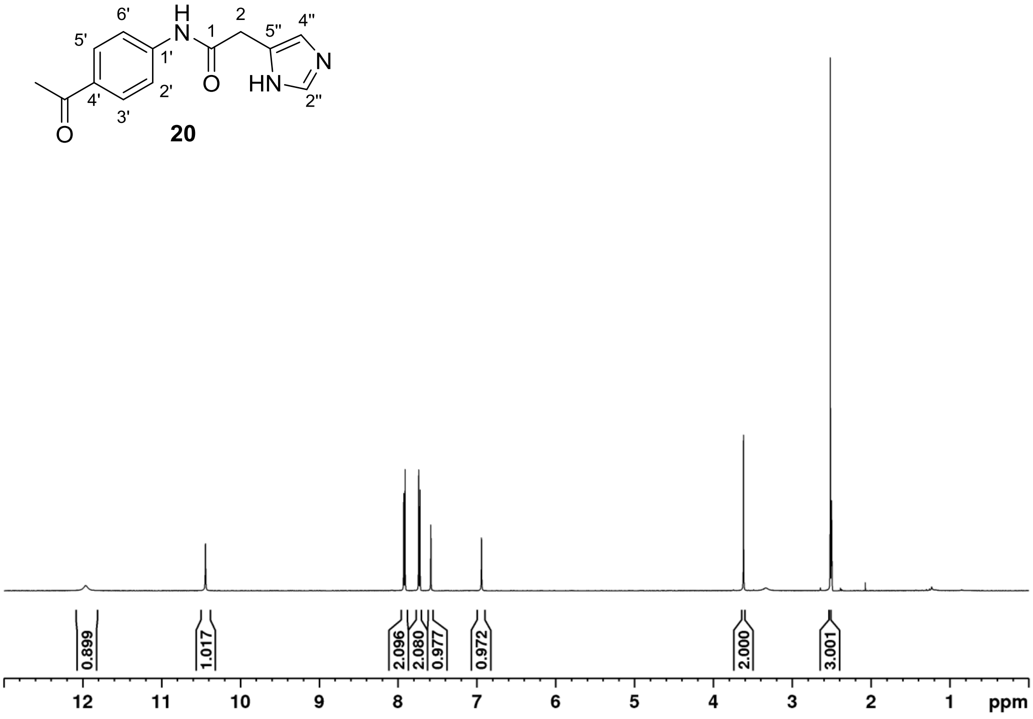


$^1$H NMR spectrum of **20** (500 MHz, DMSO-d$_6$).

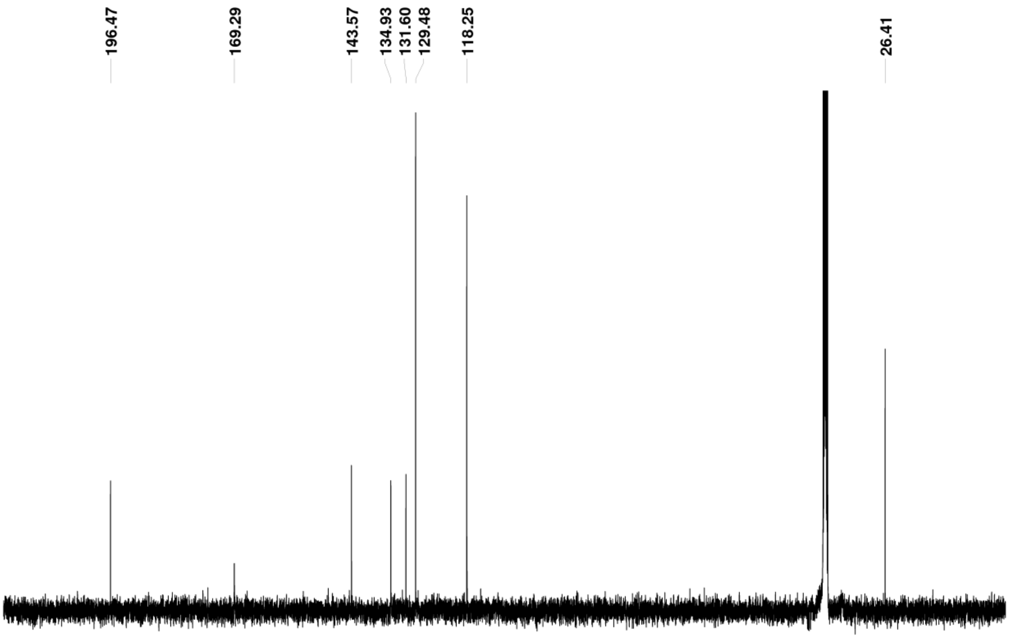


$^{13}$C NMR spectrum of **20** (126 MHz, DMSO-d$_6$).

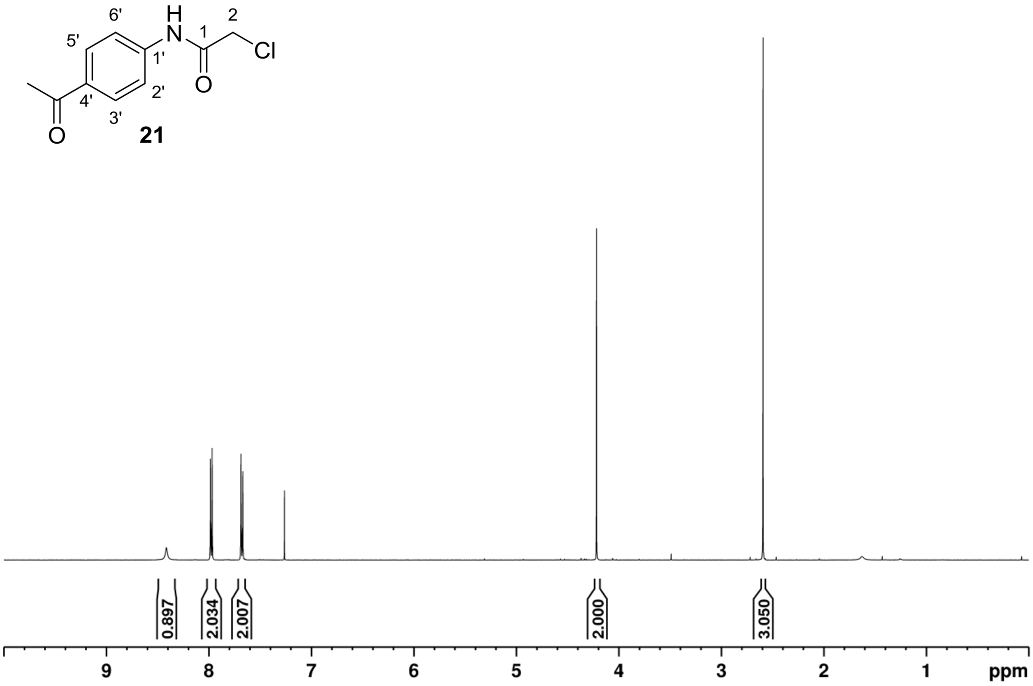


$^{1}$H NMR spectrum of **21** (500 MHz, $CDCl_3$).

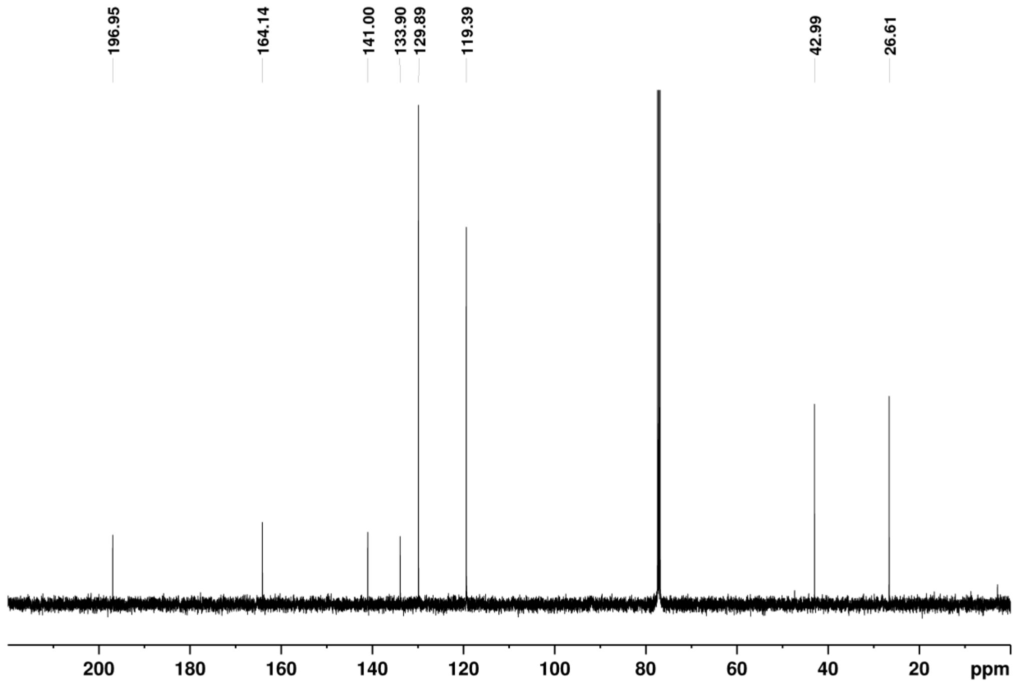


$^{13}$C NMR spectrum of **21** (126 MHz, $CDCl_3$).

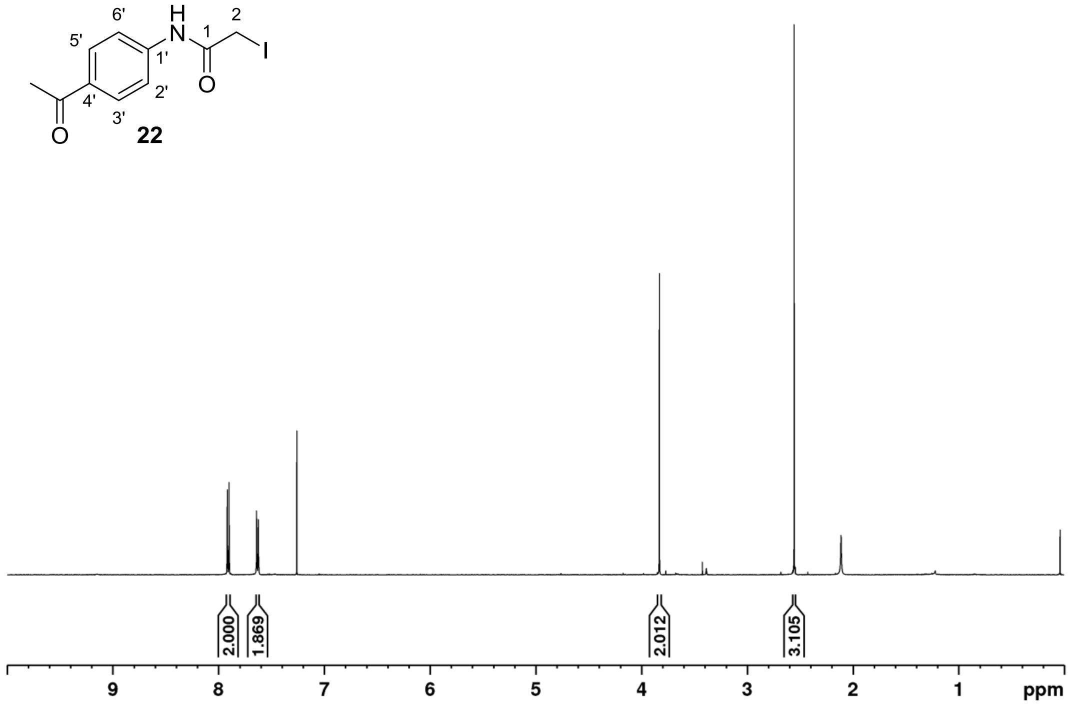


$^{1}$H NMR spectrum of **22** (500 MHz, $CDCl_3$ + $CD_3OD$).

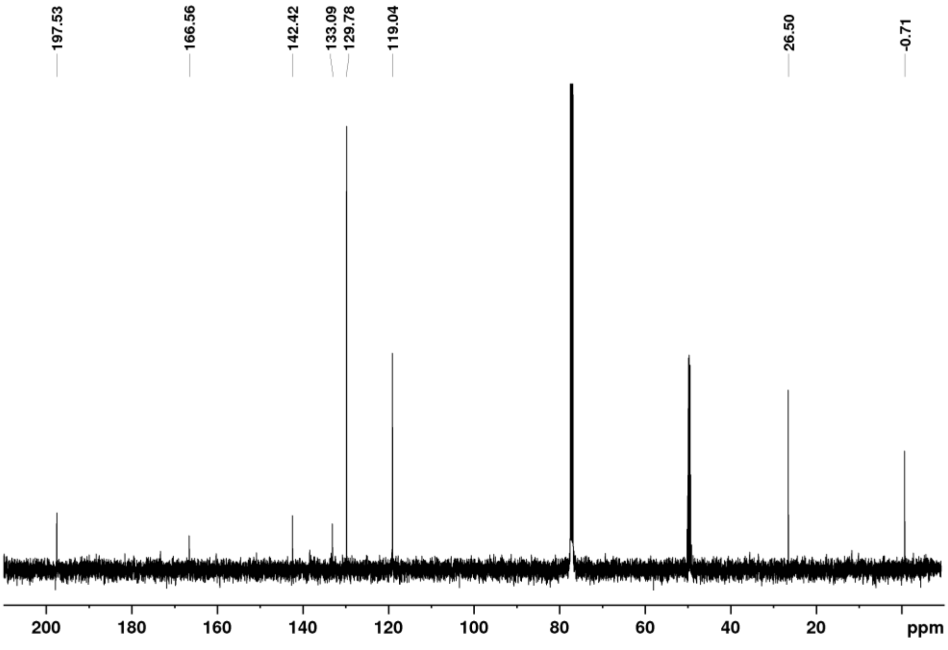


$^{13}$C NMR spectrum of **22** (126 MHz, $CDCl_3$ + $CD_3OD$).

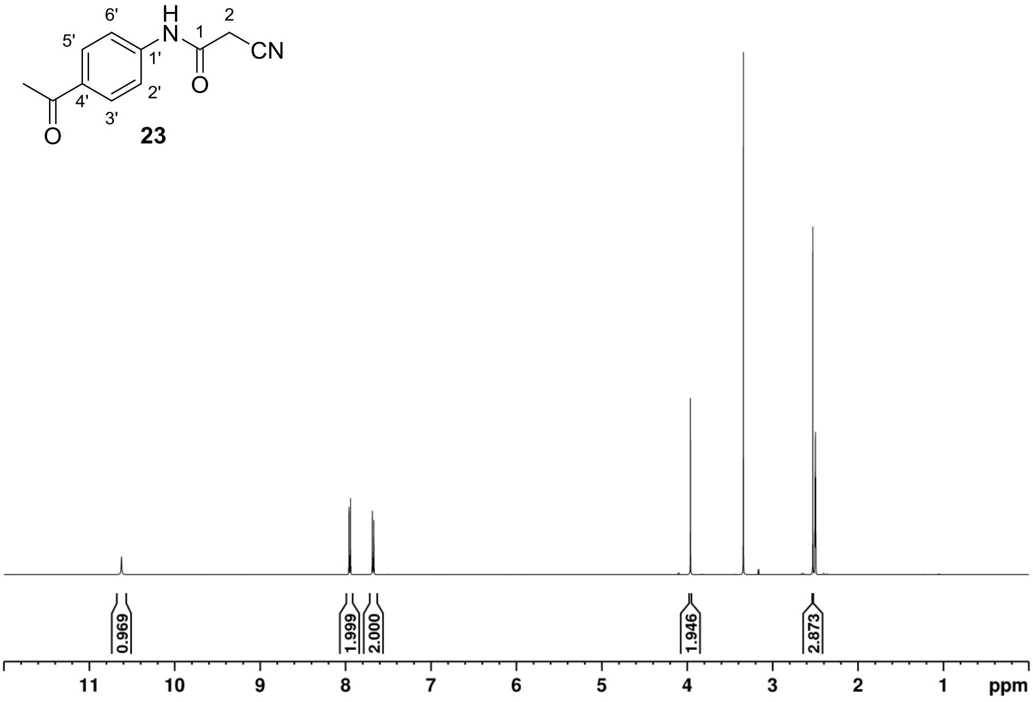


$^1$H NMR spectrum of **23** (500 MHz, DMSO-$d_6$).

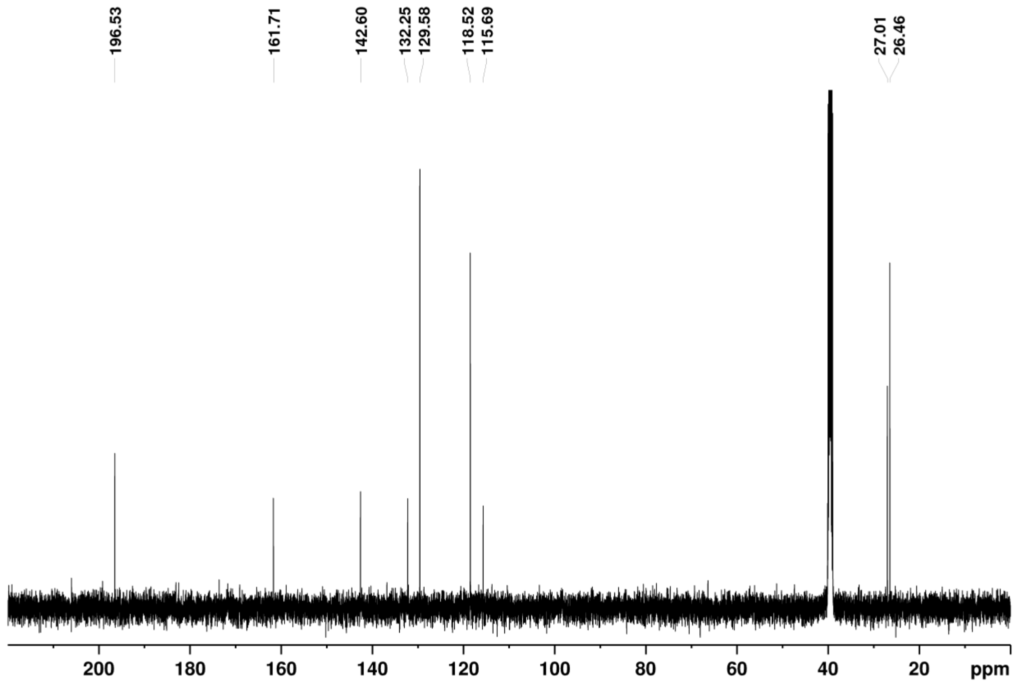


$^{13}$C NMR spectrum of **23** (126 MHz, DMSO-$d_6$).

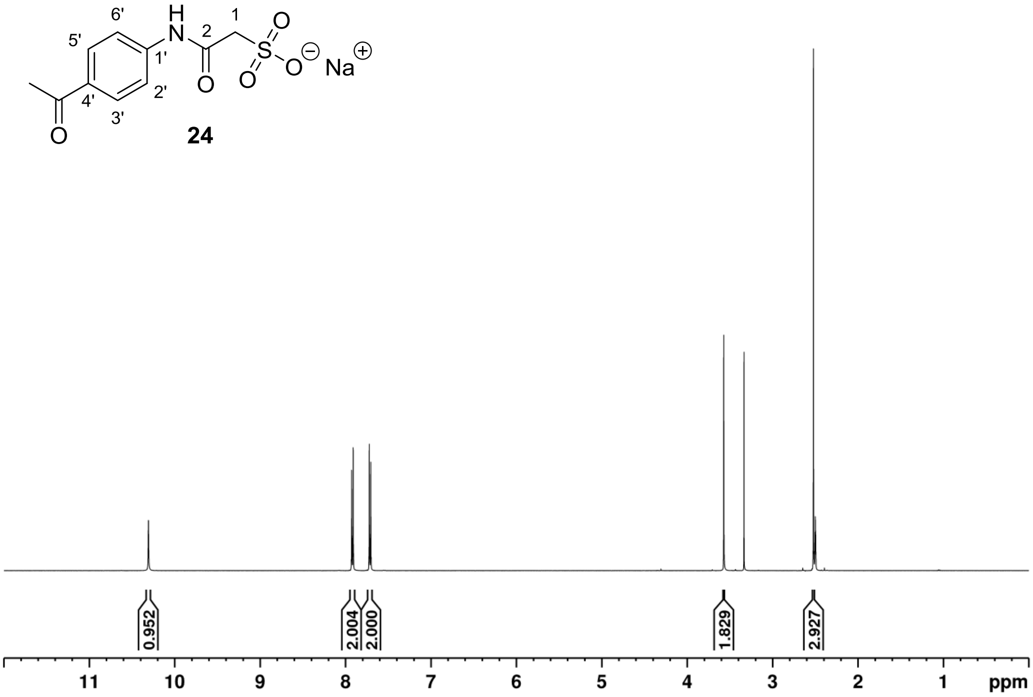


$^{1}$H NMR spectrum of **24** (500 MHz, DMSO-d$_6$).

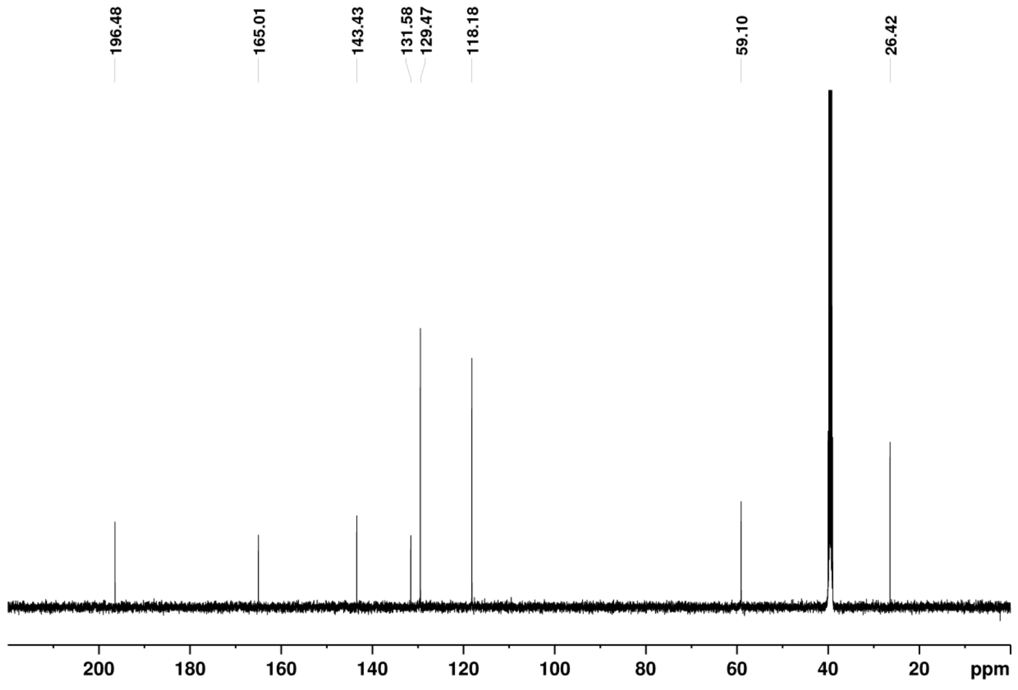


$^{13}$C NMR spectrum of **24** (126 MHz, DMSO-d$_6$).

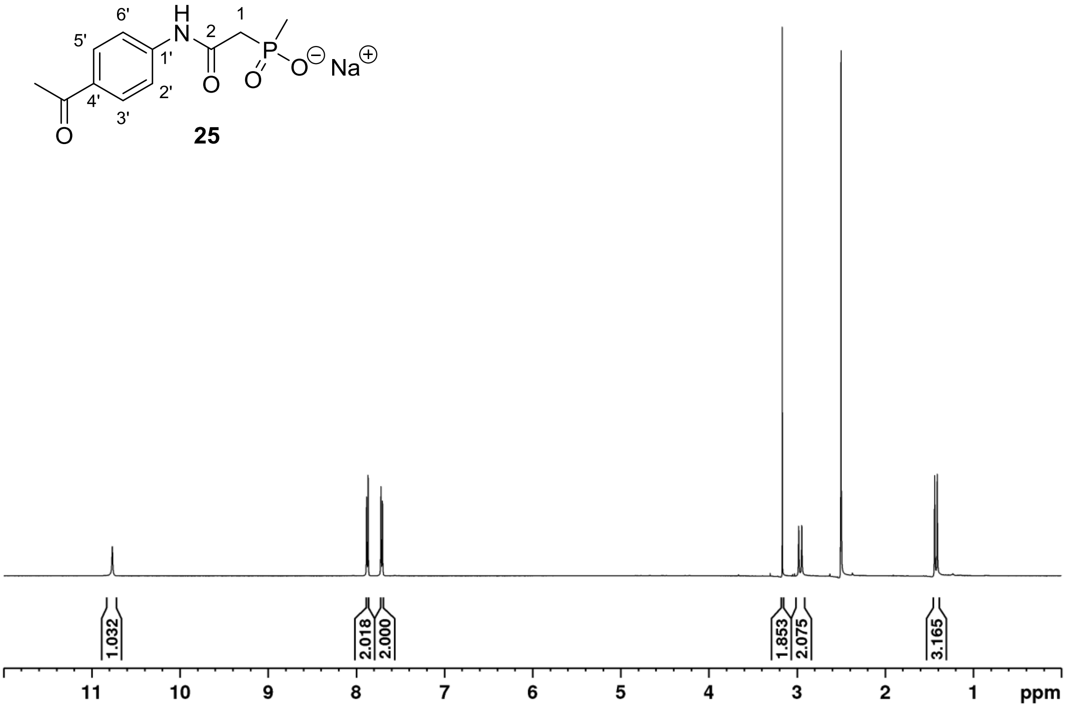


$^{1}H$ NMR spectrum of **25** (500 MHz, DMSO-$d_6$).

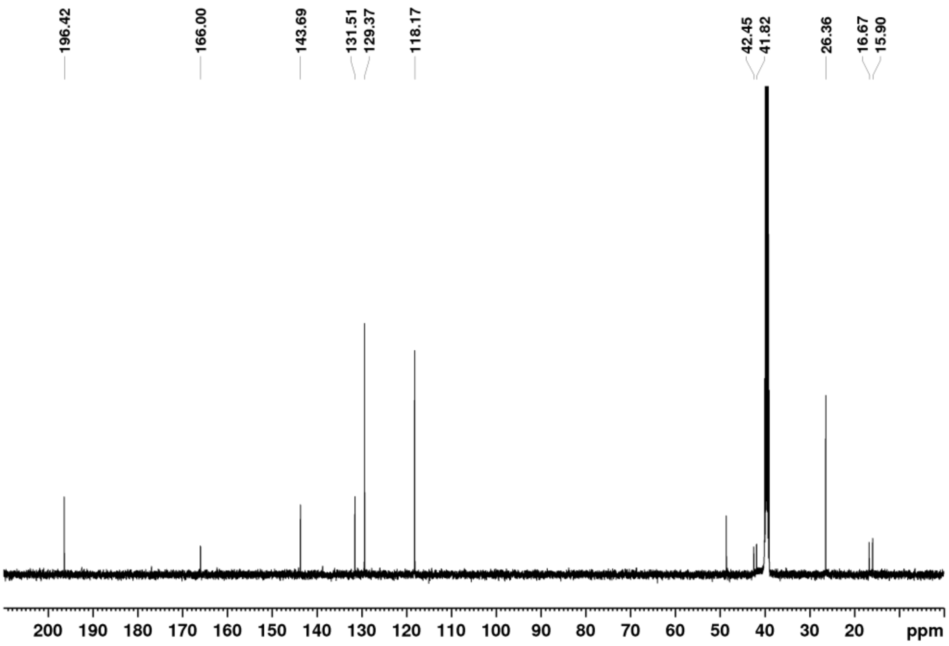


$^{13}C$ NMR spectrum of **25** (126 MHz, DMSO-$d_6$).

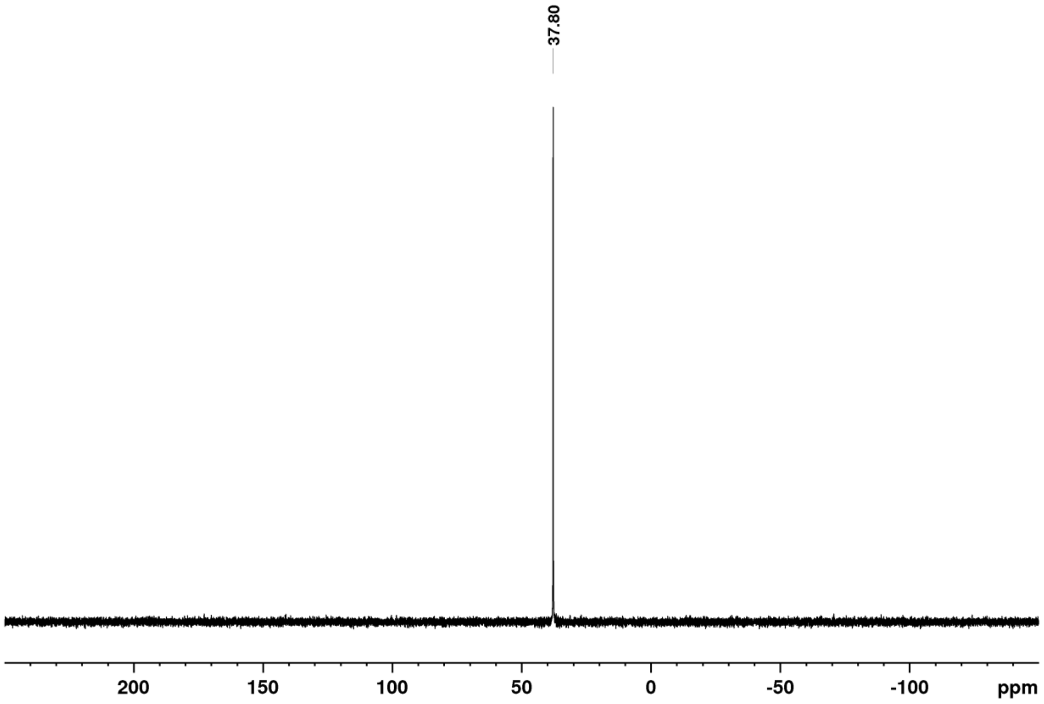


$^{31}$P NMR spectrum of **25** (162 MHz, DMSO-$d_6$).

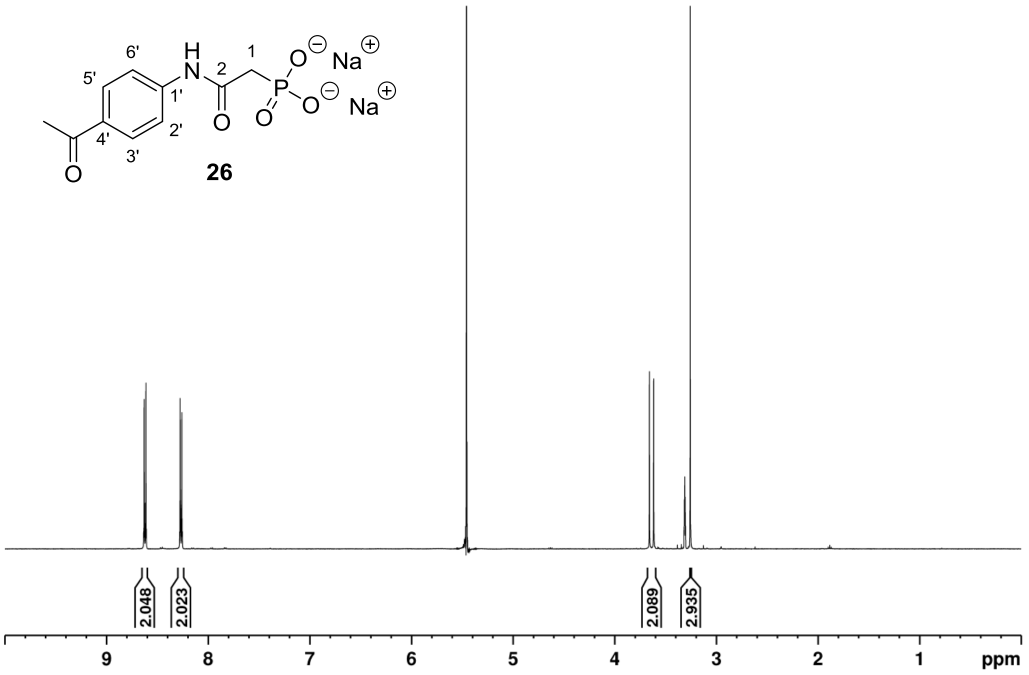


$^{1}$H NMR spectrum of **26** (500 MHz, $CD_3OD$).

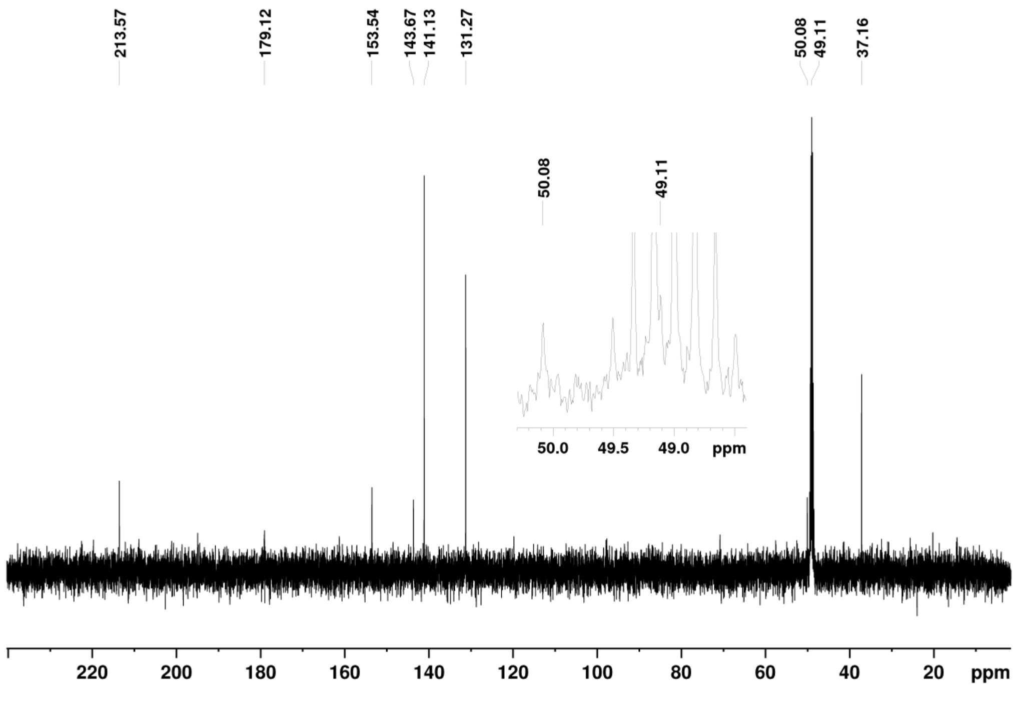


$^{13}$C NMR spectrum of **26** (126 MHz, $CD_3OD$).

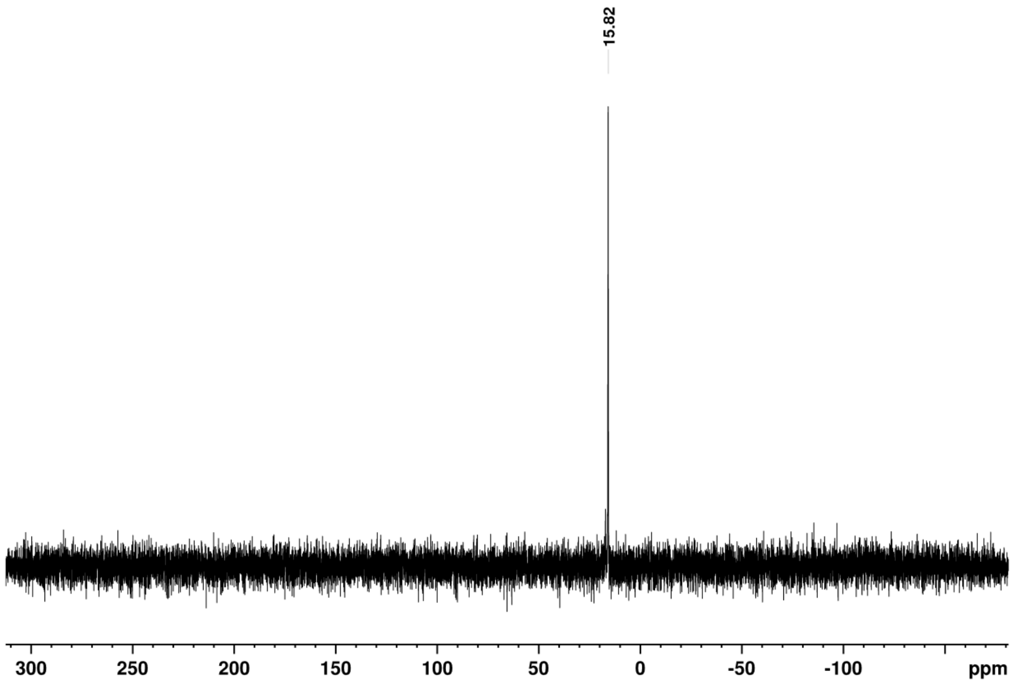


$^{31}P$ NMR spectrum of **26** (203 MHz, $CD_3OD$).

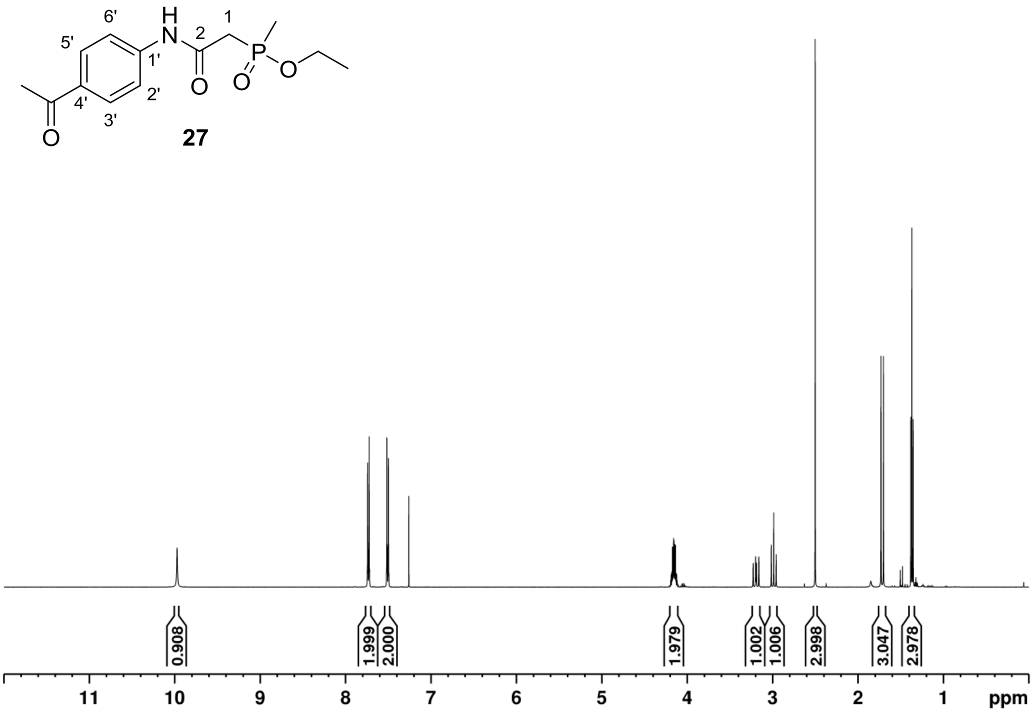


$^{1}$H NMR spectrum of **27** (500 MHz, $CDCl_3$).

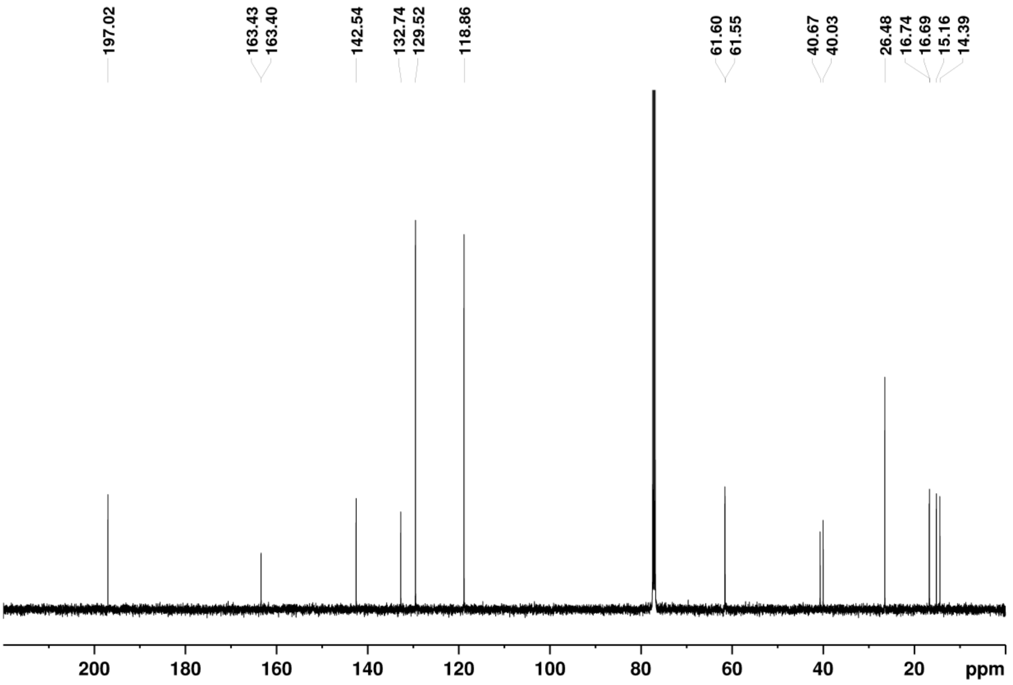


$^{13}$C NMR spectrum of **27** (126 MHz, $CDCl_3$).

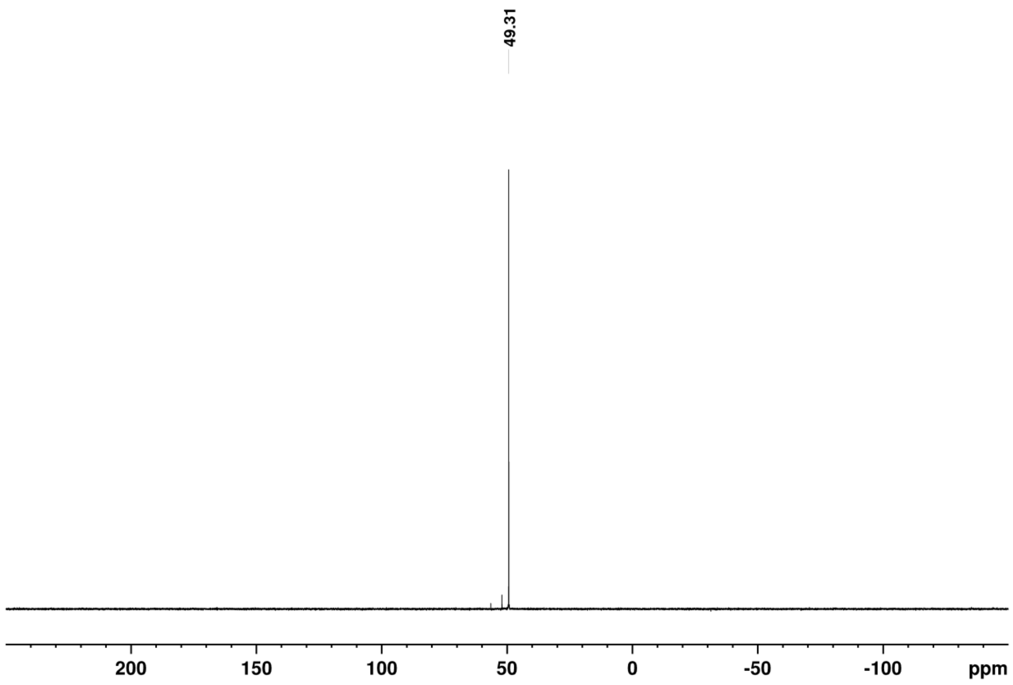


$^{31}P$ NMR spectrum of **27** (203 MHz, $CDCl_3$).

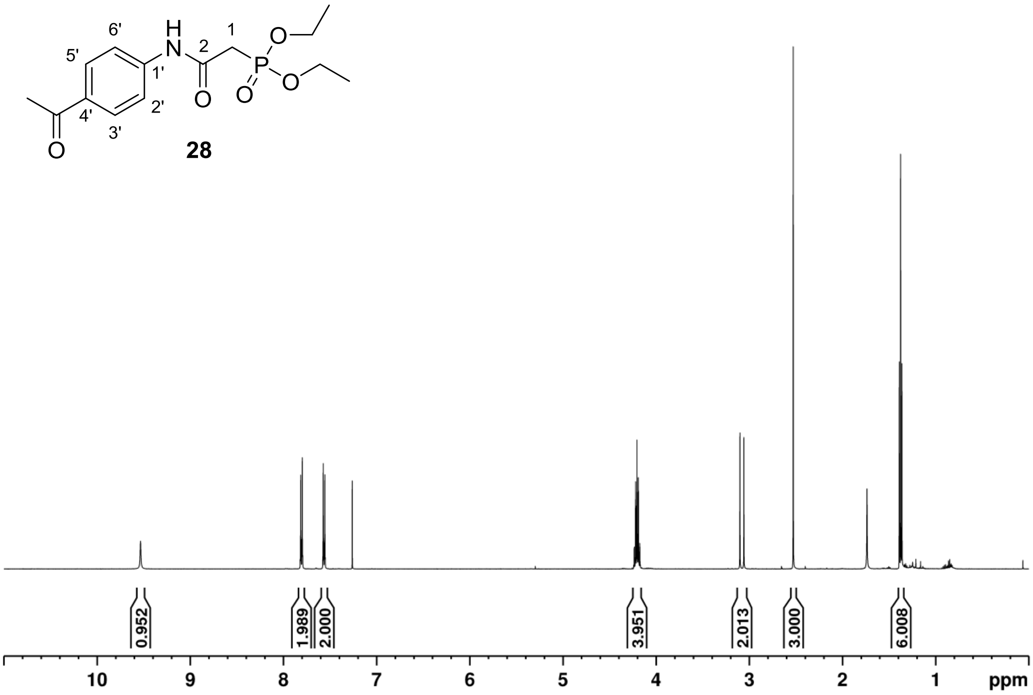


$^{1}$H NMR spectrum of **28** (500 MHz, $CDCl_3$).

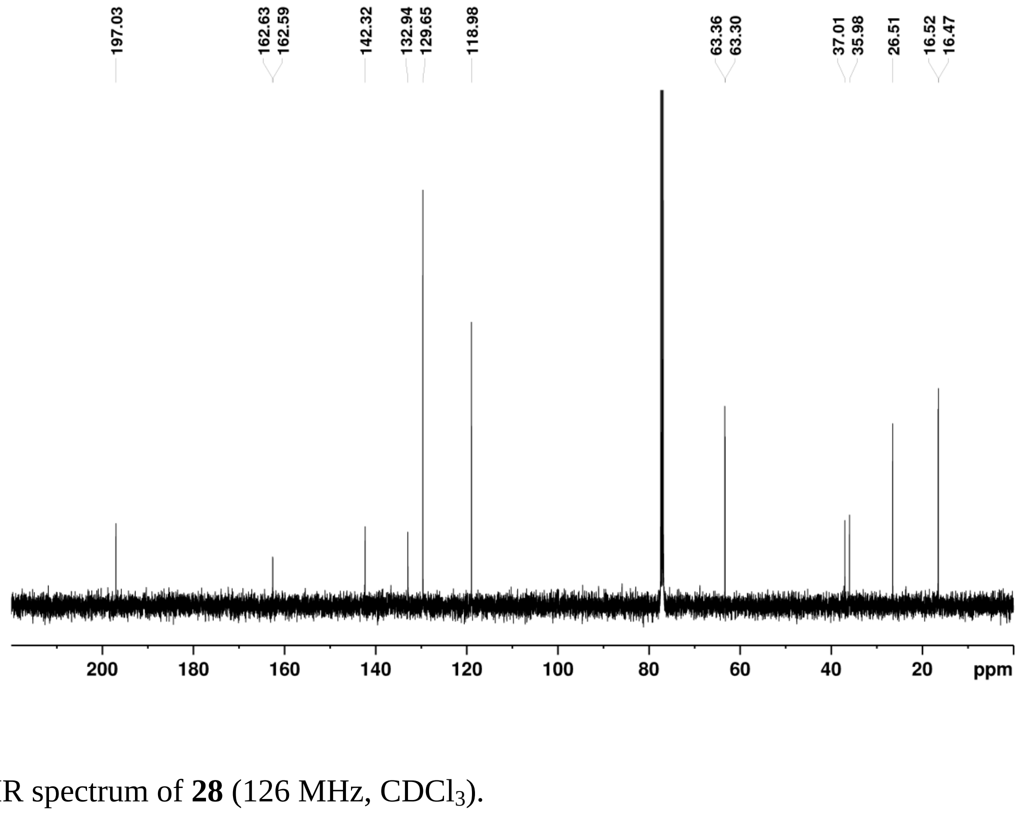


$^{13}$C NMR spectrum of **28** (126 MHz, $CDCl_3$).

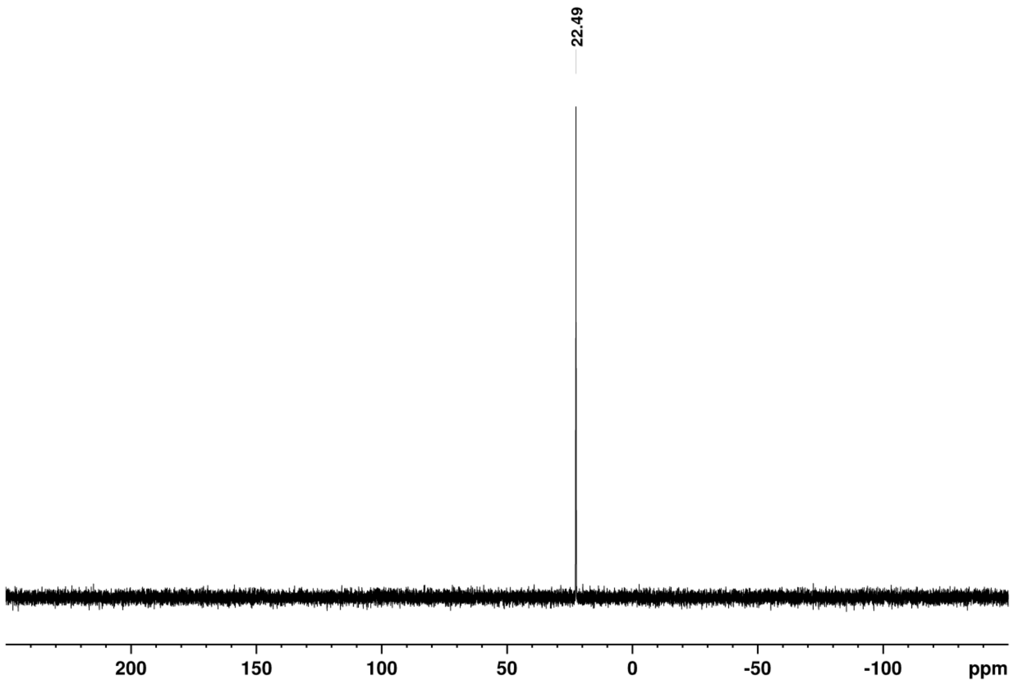


$^{31}$P NMR spectrum of **28** (203 MHz, $CDCl_3$).
